# Dihydroxyacetone decreases the dATP pool, inducing replication stress and genomic instability in BEAS-2B cells

**DOI:** 10.1016/j.jbc.2025.110876

**Published:** 2025-10-30

**Authors:** Saddam Hussain, Nayonika Mukherjee, Natalie R. Gassman

**Affiliations:** Department of Pathology, Heersink School of Medicine, University of Alabama at Birmingham, Birmingham, Alabama, USA

**Keywords:** oxidative stress, DNA damage, DNA damage response, replication stress, nucleotides, genomic instability, adenine supplementation

## Abstract

Dihydroxyacetone (DHA), found in sunless tanning products and electronic cigarette aerosol, induces cytotoxic and genotoxic effects in cells. Studies across skin and systemic models demonstrated that DHA induces cell cycle arrest and mitochondrial stress, but its impact on replication is unknown. We investigated DHA exposure effects on lung epithelial BEAS-2B cells to determine if it induces replication stress and genomic instability. Acute DHA exposure generated oxidative stress, triggering 53BP1 foci formation 24 and 48 h after exposure. Evaluation of DNA damage response showed increased levels of pChk2 and pP53, demonstrating activation of double-strand break response. Using a DNA fiber assay, we observed decreased replication fork progression, which coincided with increased micronuclei formation. Removal of DHA from cell media partially alleviated the replication stress, similar to the removal of hydroxyurea, suggesting a reversible effect. Given DHA's incorporation into glycolytic pathways and induction of mitochondrial stress, we examined its effects on nucleotide biosynthesis and pool composition. DHA exposure reduced ribonucleotide reductase (RRM1/2) expression and specifically depleted dATP pools after 48 h. We confirmed that dATP depletion drives replication stress by supplementing cells with adenine during DHA exposure, which decreased DNA lesions, reduced damage signaling, and restored replication. Adenine supplementation also partially rescued DHA-induced cytotoxicity and micronuclei formation. These data demonstrate that DHA-induced DNA damage and dATP pool depletion cause replication stress in BEAS-2B cells, providing new information on DHA's genotoxic mechanism. The inability of adenine to completely rescue micronuclei formation also suggests additional mechanisms of action that impact mitosis, requiring further investigation.

Dihydroxyacetone (DHA) is the active ingredient in most sunless tanning products and is produced by glycerol oxidation in electronic liquids of electronic cigarettes (e-cigarettes) (1, 2). With the growing popularity of sunless and spray tanning in the late 1990s, there was significant interest in the cytotoxicity and genotoxicity of DHA to the skin (3, 4, 5). These studies demonstrated that exposure to millimolar doses of DHA, relevant for tanning applications, resulted in cytotoxicity and cell cycle arrest at G2/M and produced oxidative stress and advanced glycation end products (AGEs) (4, 6). The more recent identification of DHA within the aerosol of e-cigarettes has extended the characterization of its cytotoxic and genotoxic effects beyond the skin to systemic models (7, 8, 9). These studies have confirmed the cytotoxicity of DHA at low millimolar doses in kidney, liver, lung, and cardiac cell models and demonstrated that DHA induces metabolic reprogramming, mitochondrial stress, and different classes of DNA lesions in a cell type–specific manner (7, 8, 9). More recently, it was also demonstrated that DHA was mutagenic in nontumorigenic human embryonic kidney 293T (HEK293T) cells and lung epithelial BEAS-2B cells, confirming previous mutagenic testing of DHA in *Salmonella* (3, 5).

While these works established the endpoint effects of DHA exposure in different models, they did not establish the mechanisms by which DHA induces genotoxicity or cell cycle arrest. Critically, they highlighted several differences in the induction of cell cycle arrest between cell types, which may contribute to the sensitivity of different cells to DHA and its induction of genomic instability. In the skin model work with immortalized keratinocytes, primary keratinocytes, and dermal fibroblasts, cell cycle arrest was consistently observed at G2/M, with Striz *et al.* demonstrating downregulation of G2/M DNA damage checkpoint regulation with changes in aurora kinase A, BRCA1, cyclin B1, cyclin B2, CDC25C, CDK1, CKS2, and TOP2α 24 h after 5 mM DHA (IC_20_) in human primary keratinocytes (4, 10). However, in systemic models, cell cycle arrest was not observed for the human cardiomyocyte AC16 cells; weak G1/S arrest was measured for the lung adenocarcinoma A549 cells; S-phase arrest for BEAS-2B, G2/M for HEK293T; and weak G1/S and G2/M arrest for the hepatoma HepG3 cells (5). These differences also extended to cell death mechanisms, with apoptosis observed in skin models and BEAS-2B cells, autophagy in HEK293T and A549, and a hybrid mechanism of autophagy and apoptosis in AC16 and HepG3 cells (4, 5). These results suggest that DHA metabolism produces an imbalance of metabolites or reactive species, producing cell type–specific effects and dictating genomic instability.

DHA is a three-carbon carbohydrate that can enter multiple metabolic pathways through its conversion to dihydroxyacetone phosphate (DHAP) by triose kinase FMN cyclase. Previous studies have demonstrated that DHA to DHAP conversion rates differ between tissues. DHAP can then enter into multiple metabolic pathways, with the glycolytic or glycerol pathways being the most notable (11, 12). DHAP is converted to glyceraldehyde-3-phosphate by triose phosphate isomerase and glycerol-3-phosphate by glycerol-3-phosphate dehydrogenase 1 (11, 12). Imbalances in DHA/DHAP/glyceraldehyde-3-phosphate from triose phosphate isomerase deficiency or diabetes are linked with anemia, neurological disorders, diabetes, and cancer (13). However, little is known about how these imbalances or an excess of DHA impact genomic stability and cell cycle progression. Notably, increased flux through glycolytic pathways or increased production of glycerol to reduce the excess DHA/DHAP can alter essential cofactors like ATP and NAD(P)H as well as produce reactive oxygen species (ROS) or AGEs (7, 10, 14).

Oxidative stress, AGEs, and protein carbonylation generate DNA lesions and strand breaks, contributing to genomic instability and cell cycle arrest (15, 16, 17). Single-strand break (SSB) and double-strand break (DSB) are sensed within the cells, activating DNA damage response proteins ATR or ATM. DSB response involves recruitment of MRN and 53BP1 to the damage site, activating ATM–Chk2–p53 signaling to initiate repair (18, 19, 20). The activated ATM pathway arrests the cell cycle at the G1/S phase of the cell cycle (21). SSBs recruit RPA proteins to the damage site and activate the ATR–CHK1–p21 pathway to arrest the cell cycle at the G2/M boundary (21, 22, 23, 24). Failure to repair the DNA damage results in mitotic defects, chromosomal aberrations, mutagenesis, and cell death (25, 26).

Beyond induced DNA damage, metabolic changes in ATP, nucleotides, and essential cofactors can lead to replication stress (27). One essential component of DNA replication is nucleotide pool supply during the S phase of the cell cycle (28, 29). Nucleotide pools vary during different phases of the cell cycle (30). The demand for nucleotides during the S phase increases several fold compared with the G1 phase, requiring increased availability of the nucleotide pool (31). The increased supply of dNTPs is regulated through *de novo* and salvage nucleotide synthesis pathways and an increased activity of protein ribonucleotide reductase (RNR) (32, 33). High RNR activity is maintained during S phase (34). Mild imbalances in the nucleotide pool during S phase can promote mutagenesis and replication stress and activate intra-S phase checkpoint in *Saccharomyces cerevisiae* (35, 36). During dNTP biosynthesis, the RNR enzyme is reduced by thioredoxin, which is further reduced in the presence of NAD(P)^+^/NAD(P)H to regenerate oxidized RNR (37). NAD(P)^+^/NAD(P)H are required during glycolysis, the Krebs cycle, and nucleotide metabolism, and perturbations in the NAD(P)^+^/NAD(P)H pool during nucleotide metabolism have been shown to result in replication stress in the cells (38).

Despite the intersection of DHA with the essential cofactors for nucleotide synthesis and cell cycle progression, few studies have focused on understanding the mechanisms underlying the potential replication stress induced by DHA and its relationship with DHA's genotoxicity. DHA exposure altered mitochondrial metabolism and NAD(P)^+^/NAD(P)H redox balance in H9c2, HEK293T, and HepG3 cells (7, 8, 9). Mitochondria are the primary site for ATP generation and are also involved in the biosynthesis of nucleotides. Defects in mitochondrial function and metabolism are associated with DNA damage, replication stress, and cell death. We previously demonstrated that acute doses of DHA produced a mixture of DNA lesions, with oxidative lesions and DNA crosslinks being predominant in liver, lung, and cardiac cells (5). If unrepaired, these types of DNA damage produce transcription-replication conflicts, activating DNA damage response and inducing cell cycle arrest (39, 40). Moreover, cytotoxic doses of DHA produce varied effects on proteins related to DNA damage response, including cleaved PARP, 53BP1 foci formation, and p21 protein expression. BEAS-2B cells showed oxidative lesions, a marked increase in 53BP1 foci formation, and a depletion in p21 expression (5).

Even though these studies indicated the presence of DNA damage, cell cycle arrest, and defects in mitochondrial metabolism, whether it induces DNA damage response, replication stress, and genomic instability remains unexplored. Here, we examine the acute exposure effects of DHA on BEAS-2B cells and focus on the replication effects of exposure.

## Results

### DHA induced a mixture of DNA lesions and protein carbonylation

**Figure 1:**
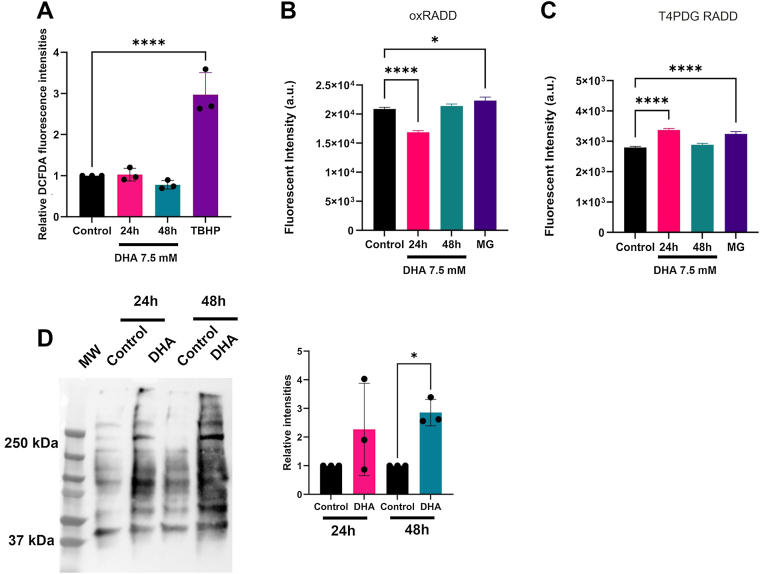
**DHA exposure results in DNA lesions and protein carbonylation.***A,* relative DCFDA fluorescence intensities from BEAS-2B cells exposed to 7.5 mM DHA and 250 μM TBHP for 24 and 48 h, respectively. *B,* measurement of oxidative DNA lesions using oxRADD after exposure to 7.5 mM DHA for 24 and 48 h. Cells were also exposed to 25 μM methylglyoxal (MG) as a positive control. *C,* measurement of crosslink-type DNA lesions using T4PDG RADD after exposure to 7.5 mM DHA for 24 and 48 h. *D,* immunoblot of protein carbonylation in DHA-exposed BEAS-2B cells at 24 and 48 h. Quantification of protein carbonylation levels from the three biological repeats. Graphs are displayed as the mean fluorescence intensity ± SEM over three biological replicates. The statistical difference was analyzed by one-way ANOVA and Dunnett's post hoc test and displayed as follows: ∗*p* < 0.05 and ∗∗∗∗*p* < 0.0001. DCFDA, dichlorodihydrofluorescein diacetate; DHA, dihydroxyacetone; oxRADD, oxidative repair assisted damage detection; TBHP, tert-butyl hydrogen peroxide; T4PDG, T4 pyrimidine dimer glycosylase.

Previous work with DHA in the skin and systemic models used high cytotoxic doses to determine genotoxic effects (4, 5, 6). These doses can make it difficult to differentiate genotoxic mechanisms. In the skin, there were inconsistent findings about the generation of ROS and induction of DNA damage independent of cell death (6, 9, 14). In the systemic models, we found little cytosolic ROS generation despite measuring increased levels of oxidative DNA lesions (5). Therefore, we evaluated the generation of ROS and DNA lesions in BEAS-2B cells dosed with its IC_50_ dose (7.5 mM). Cells were exposed to 7.5 mM DHA for 24 and 48 h, and intracellular ROS was measured using CM-H_2_ dichlorodihydrofluorescein diacetate (DCFDA) (Fig. 1*A*). Tert-butyl hydrogen peroxide, a known ROS inducer, was used as a positive control. DHA did not produce significant levels of ROS in the BEAS-2B cells (Fig. 1*A*).

We then confirmed the presence of DNA lesions using the DNA adduct detection method, repair assisted damage detection (RADD). RADD detects DNA adducts using bacterial DNA glycosylases to excise adducts from the DNA. The gapped DNA or strand breaks resulting from the excision of the DNA adducts are then filled using Klenow exo-, which inserts a tagged dUTP for fluorescent detection of the lesion site (41). We used specific cocktails of the DNA glycosylases to detect oxidative lesions (Fapy-DNA glycosylase [FPG] + endonuclease VIII [endo VIII] + endo VI, oxRADD) and pyrimidine crosslinks (T4 pyrimidine dimer glycosylase [T4PDG] + endonuclease IV [endo IV], T4PDG RADD) (Fig. 1*B*). We used 25 μM methylglyoxal (MG) exposed for 24 h as a positive control for inducing DNA damage because DHA is predicted to produce MG and AGEs (14, 42). BEAS-2B cells dosed with 7.5 mM DHA for 24 h showed a decrease in oxidative DNA lesions, which indicates stimulated DNA repair of these lesions (Fig. 1*B*). At 48 h, the oxidative lesions returned to basal levels. We also observed increased DNA crosslinked lesions 24 h after exposure to DHA (Fig. 1*C*).

We also assessed uracil (UDG + endo IV, UDG RADD) and alkylated lesions (AAG + endo IV, AAG RADD) even though these lesions were not elevated at 12.5 mM doses (Fig. S1, *A* and *B*) (5). Uracil lesions increased and remained significantly elevated 48 h after exposure. We did not observe any significant changes in alkylated DNA lesions. MG exposure produced elevated oxidative and crosslinked lesions and stimulated DNA repair for uracil and alkylated lesions (Fig. 1, *B* and *C*, Fig. S1, *A* and *B*). This profile is distinct from that induced by DHA, with the oxidative and uracil lesions showing the most significant difference in damage levels. These results suggest that DHA produces reactive metabolites besides MG, which have a different spectrum of lesions and DNA repair effects. Because DHA potentially forms adducts and reacts with amine groups of proteins through a Maillard reaction, we also measured protein damage caused by DHA by protein carbonylation. Twenty-four hours after DHA exposure, BEAS-2B cells showed increased protein carbonylation that significantly increased 48 h after treatment (Fig. 1*D*).

We previously observed elevated crosslink-type lesions at the IC_90_ DHA (12.5 mM), and the structure of these lesions is unclear (5). T4PDG predominantly recognizes UV-induced cyclobutane pyrimidine dimers (CPDs) and 6-4-photoproducts. Therefore, we tried to characterize the lesion type using a slot blot assay. DNA was extracted from BEAS-2B cells exposed to DHA for 24 and 48 h to perform an immunoslot blot using anti-CPD antibody. DNA exposed to 400 J/m^2^ UV was used as a positive control. Compared with the control, DHA treatment showed no changes in CPD formation at 24 h (Fig. S1*C*). Exposure to DHA for 48 h increased DNA CPD formation, similar to the CPD observed in UV-treated DNA. While the increased DNA CPD levels are not significant, they are consistent with the increased T4PDG type of DNA lesions, suggesting that DHA exposure for 48 h forms CPD-like dimers in the BEAS-2B cells. Given the elevated uracil lesions, we also measured the expression of UNG1 and UNG2 in the BEAS-2B cells using immunoblotting at 24 and 48 h after DHA exposure. No significant change in UNG1 or UNG2 levels was observed after DHA exposures (Fig. S1*D*). We also examined the protein expression levels of DNA repair proteins within the base excision repair and nucleotide excision repair pathways but did not observe any significant changes in protein levels along these pathways (Fig. S2). These observations demonstrate that DHA exposure generates complex DNA lesions and protein damage through carbonylation in BEAS-2B cells.

### DHA produced 53BP1 foci and activated DNA DSB response

**Figure 2:**
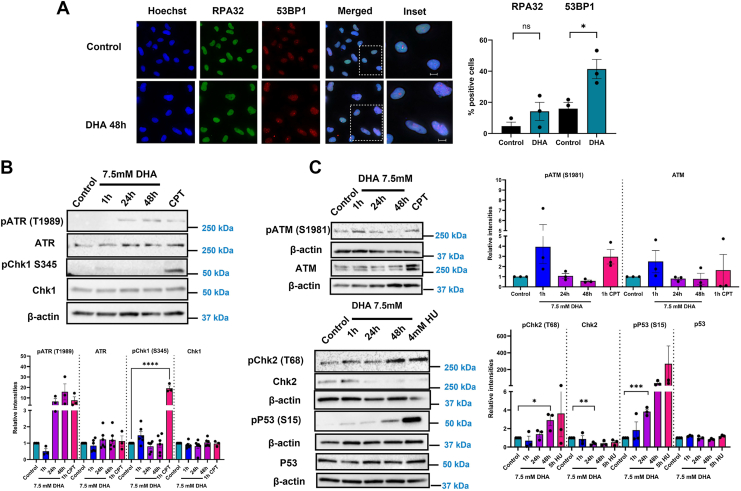
**DHA treatment leads to 53BP1 foci formation and activation of DNA damage response.***A,* RPA32 and 53BP1 foci formation observed in DHA-treated BEAS-2B cells 48 h after DHA treatment. Cells with a minimum of six foci are considered as damage positive. The scale bar represents 12.5 μm. *B,* ATR-mediated DNA damage response proteins probed in DHA-treated cells at 1, 24, and 48 h. Hydroxyurea (HU) is used as a positive control with cells exposed at 4 mM for 5 h. *C,* ATM-mediated DNA damage response proteins probed after DHA treatment. Camptothecin (CPT) and HU are used as positive controls with cells treated at 100 nM for 1 h or 4 mM for 5 h, respectively. Graphs are displayed as the mean intensity ± SEM over three biological replicates. The statistical difference was analyzed by one-way ANOVA coupled with Dunnett's post hoc test and displayed as follows: ∗*p* < 0.05, ∗∗*p* < 0.01, and ∗∗∗*p* < 0.001. DHA, dihydroxyacetone.

DNA lesions obstruct the movement of transcription and replication assemblies. Failure to repair the DNA lesions leads to DNA strand breaks, activating DNA damage response. We examined if DHA-induced lesions or protein damage activated DNA damage response. BEAS-2B cells exposed to 7.5 mM DHA for 48 h showed increased RPA32 and 53BP1 foci formation (Fig. 2*A*). RPA32 binds to the single-stranded DNA, and the number of foci-positive cells increased, though not significantly, in DHA-treated cells. We confirmed the lack of RPA32 involvement by measuring the protein levels of pRPA32(S33) and total RPA32 in cell lysates (Fig. S3*A*). We observed only a slight increase in the ratio of pRPA32–RPA32 in DHA-treated cells as compared with the camptothecin (CPT)-treated positive control (Fig. S3*B*). Next, we checked 53BP1, which is recruited to DSB, and observed a significant increase in 53BP1 foci–positive cells, suggesting DSBs after DHA exposure (Fig. 2*A*).

As DNA damage response activates ATR and/or ATM pathways (SSB or DSB sensing, respectively), we examined the activation of these pathways using immunoblotting at 1, 24, and 48 h after DHA exposure (Fig. 2, *B* and *C*). Cells were also treated with CPT or hydroxyurea (HU) as a positive control for 1 and 5 h, respectively, to induce replication stress–associated DNA damage as a positive control. CPT is a topoisomerase I inhibitor, whereas HU is an inhibitor of RNR, which converts nucleotide diphosphates to deoxynucleotide diphosphates for DNA replication. HU treatment for shorter times suppresses replication fork progression as opposed to the longer treatment, where the replication fork stalls and cannot restart replication, resulting in fork collapse (43). DHA exposure increased phosphorylated ATR (pATR T1989) but not total ATR levels, consistent with RPA32 foci results and similar to the levels observed in HU-treated cells (Fig. 2*B*). Despite the increase in pATR T1989 levels, DHA exposure showed no change in pChk1 S345 and total Chk1 protein levels (Fig. 2*B*). We only observed a slight increase in the ratio of pChk1 S345/Chk1 at 1 h of DHA exposure (Fig. S3*D*), suggesting that downstream single-strand DNA damage signaling is inactive. Since ATR could be activated on other residues such as S428 in response to single-strand breaks, we also examined the phosphorylation at this site in DHA-exposed cells. Compared with pATR T1989 levels, there was only a slight increase in pATR S428 (Fig. S3*C*), with no change in pChk1 S345 levels, confirming minimal activation of the SSB pathway. Evaluation of the DSB response revealed an increase in pATM levels and total ATM (Fig. 2*C*). The ratio of pATM S1981/ATM increased with DHA exposure at 1 and 48 h (Fig. S3*E*), indicating activation of the DSB response. As expected, the downstream effector kinase of ATM protein, pChk2, showed a gradual increase starting 24 h after DHA exposure and peaking at 48 h (Fig. 2*C*). The total Chk2 levels also decreased in DHA-treated cells, consistent with the HU-treated samples. As pChk2 activates p53 on S15 during DSB response, we examined pP53 and total p53 levels in DHA-treated cells. DHA exposure led to a significant increase in pP53 levels 1 h after exposure. Total p53 levels were unchanged, suggesting the activation of p53 after DHA treatment (Fig. 2*C*). These data suggest that DHA exposure activated the DSB response through the ATM–Chk2–p53 pathway.

### DHA exposure altered cell cycle progression at S phase and decreased the expression of replication proteins Bloom's syndrome and Werner

**Figure 3:**
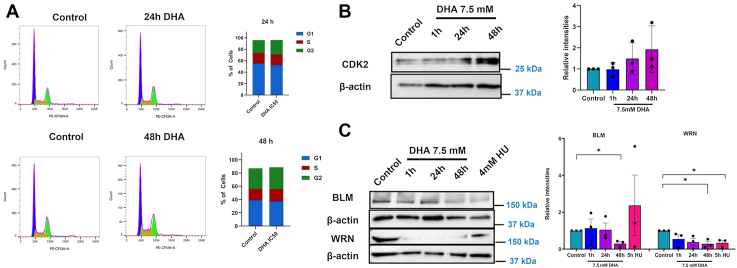
**DHA exposure alters cell cycle progression at the S phase.***A,* cell cycle analysis of DHA-treated BEAS-2B cells 24 and 48 h after treatment. Representative cell cycle analysis graphs are displayed for each time point, with the mean percentage over three biological replicates in the bar graphs. *B,* immunoblot analysis of S phase CDK2 protein after DHA treatment for 1, 24, and 48 h. *C,* immunoblot analysis of replication stress proteins BLM and WRN probed in DHA-treated BEAS-2B cells. HU is taken as a positive control. Graphs are displayed as the mean intensity ± SEM over three biological replicates. The statistical difference was analyzed by one-way ANOVA and Dunnett's post hoc test and displayed as follows: ∗*p* < 0.05, ∗∗*p* < 0.01, and ∗∗∗*p* < 0.001. BLM, Bloom's syndrome; DHA, dihydroxyacetone; HU, hydroxyurea; WRN, Werner.

DNA damage during the S-phase of the cell cycle could suppress cell cycle progression to repair the DNA and maintain genomic integrity. As we observed the activation of DNA damage response in DHA-treated cells, we asked whether it leads to cell cycle arrest. Previously, when BEAS-2B cells were treated with an IC_90_ dose of DHA (12.5 mM), there was partial S phase arrest. BEAS-2B cells treated with 7.5 mM DHA dose showed no changes in cell cycle phases after 24 or 48 h. There were slight changes in G1 phase after 24 h (42.0 ± 6.73 *versus* 38.57 ± 7.18) with no changes in S phase (15.97 ± 1.56 *versus* 16.20 ± 1.66) and G2/M (31.67 ± 4.9 *versus* 32.30 ± 3.74). At 48 h, cells showed an increase in S (6.77 ± 1.94 *versus* 18.30 ± 3.10) and no changes in G1 (39.13 ± 5.56 *versus* 37.23 ± 5.68) and G2/M (31.07 ± 3.73 *versus* 33.07 ± 4.45) (Fig. 3*A*). We also examined the expression of CDK2, which is required to progress through S phase. We observed increased CDK2 expression 24 and 48 h after DHA exposure (Fig. 3*B*), indicating some disruption in S phase progression.

DNA lesions can cause replication fork stalling, extending the time in S phase. Prolonged fork stalling may lead to fork collapse and formation of DSB, explaining the increase in 53BP1 foci observed. Werner (WRN) and Bloom's syndrome (BLM) proteins are recruited to stalled replication forks to promote repair and recovery (44). We examined if BLM and WRN protein levels are altered after DHA exposure (Fig. 3*C*). BLM was unchanged at 1 and 24 h but showed a steep decrease at 48 h of DHA exposure. WRN also showed a significant decrease in expression starting 1 h after DHA exposure, which continued out to 48 h of DHA treatment. We compared the protein levels of BLM and WRN in DHA-treated cells to 4 h treatment with HU, and the protein trends for BLM but not WRN are distinct from HU, though a decrease in these proteins is observed with HU treatment (Fig. 3*C*). These data demonstrate that DHA altered S-phase progression and promoted the loss of WRN similar to the HU-treated cells.

### BEAS-2B cells showed replication stress and genomic instability after DHA treatment

**Figure 4:**
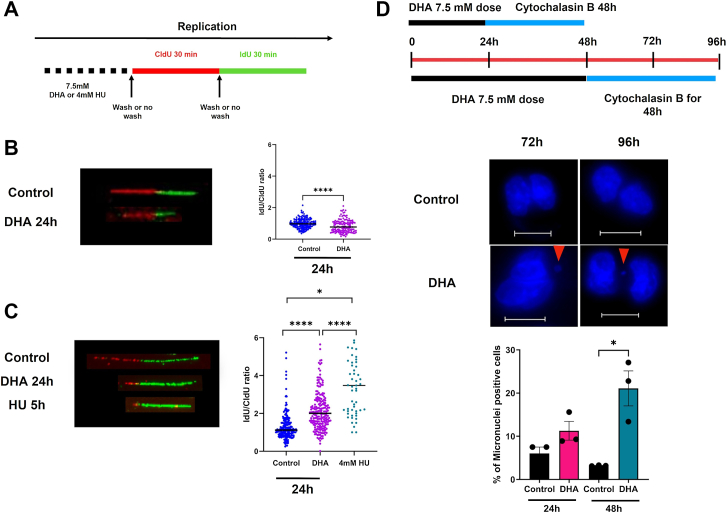
**DHA treatment results in replication stress and genomic instability in BEAS-2B cells.***A,* schematic of DHA treatment for fiber assay. *B,* representative fiber images and quantification of DNA fiber for control, DHA-treated, and HU-treated BEAS-2B cells labeled with 100 μM CldU and IdU (*red* and *green labels*, respectively) for 30 min. Fiber lengths represent arbitrary units measured from >150 fibers. *C,* representative fiber images and quantification of DNA fiber for control, DHA-treated then washed, and HU-treated BEAS-2B cells labeled with CldU and IdU for 30 min. Fiber lengths represent arbitrary units measured from >150 fibers. *D,* schematic of cytokinesis block micronuclei assay in the presence of 7.5 mM DHA and 3 μg/ml cytochalasin B treatment. Hoechst staining of nuclei to show micronuclei formation in the presence of DHA. The scale bar represents 12.5 μM. Graphs are displayed as the mean ± SEM over three biological replicates. The statistical difference was analyzed by one-way ANOVA coupled with Dunnett's post hoc test or Student's *t* test and displayed as follows: ∗*p* < 0.05, ∗∗*p* < 0.01, and ∗∗∗*p* < 0.001. DHA, dihydroxyacetone.

With the observations that DHA activated DSB damage signaling, prolonged the S phase, and decreased expression of replication proteins BLM and WRN, we hypothesized that DHA induced replication stress in BEAS-2B cells. Replication stress can decelerate replication fork movement, induce stalling, or promote the collapse of the replication assembly. DNA combing is the standard method to measure fork progression through the sequential addition of thymidine analog 5-chloro-2′-deoxyuridine (CldU) and 5-iodo-2′-deoxyuridine (IdU) followed by immunodetection of labeled DNA fibers. BEAS-2B cells were treated with a 7.5 mM dose of DHA for 24 h, followed by the addition of CldU and IdU for 30 min, respectively, in the continued presence of DHA or following removal of DHA by washing (Fig. 4*A*). We continued to use treatment with 4 mM HU for 5 h as a positive control. Immunolabeled DNA fibers were imaged, and the length of each label on the fibers was measured to show the relative ratio of IdU–CldU incorporation.

BEAS-2B cells were exposed to DHA for 24 h or mock treated (Fig. 4*A*). Mock-treated control cells showed an IdU–CldU ratio of 1.0 ± 0.02 (Fig. 4*B*). When DHA was left in the cell media during thymidine analog addition, we observed suppressed nucleotide analog incorporation at the replication fork as indicated by the decreased lengths of the CldU- and IdU-labeled tracks. The ratio of IdU–CldU was 0.83 ± 0.03 compared with the control (Fig. 4*B*). DHA inhibited the incorporation of the nucleotide analogs, impeding the progression of the replication fork and promoting replication stress.

We then tested the removal of DHA before adding the thymidine analogs. Washing the DHA away before CldU addition showed a further reduction of the CldU incorporation within the 30 min pulse (Fig. 4*C*). However, the IdU track recovered to the levels of the mock-treated cells. The resulting ratio of IdU–CldU was 2.27 ± 0.09 (*p* < 0.0001) compared with the control 1.29 ± 0.05, similar to the nucleotide analog incorporation ratio observed in cells exposed to 4 mM HU for 5 h (IdU–CldU = 3.8 ± 0.30, *p* < 0.0001) (Fig. 4*C*). HU induces a reversible effect on the replication fork, and its removal results in the restart of stalled forks and cell cycle progression. Therefore, our data indicate that DHA-induced replication stalling is partially alleviated by DHA's removal, similar to HU.

Replication stress is linked to chromosomal aberrations and micronuclei formation. DHA exposures at 12.5 mM DHA induced chromosomal aberrations in the BEAS-2B cells (5), possibly related to the observed replication stress. Therefore, we used a cytokinesis-block micronuclei assay to determine if DHA-induced replication stress led to micronuclei formation. BEAS-2B cells were treated with 7.5 mM DHA for 24 or 48 h, and then cytochalasin B, a cytokinesis inhibitor, was spiked in the cell culture media with DHA still present (Fig. 4*D*). Cells were incubated for 48 h (nearly two cell cycles) in cytochalasin B-containing media before staining with Hoechst. DHA treatment showed increased micronuclei formation compared with the mock-treated control cells for both time points (Fig. 4*D*). However, the number of micronuclei was significantly increased after 48 h of DHA (96 h final endpoint), demonstrating genomic instability (Fig. 4*D*).

### dATP pool is altered in DHA-treated BEAS-2B cells

**Figure 5:**
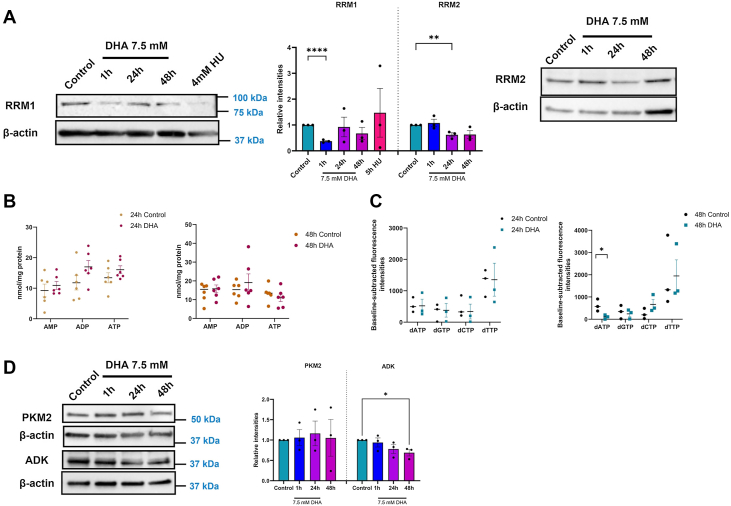
**dATP pool is altered after DHA treatment in BEAS-2B cells.***A,* immunoblot analysis of RNR protein subunit RRM1 and RRM2 showing decreased expression when exposed to DHA and HU for indicated times. *B,* quantification of adenosine pool through LC–MS 24 and 48 h after DHA treatment. *C,* measurement of dNTP pool using a quantitative PCR–based assay at 24 and 48 h after DHA treatment revealed depletion of the dATP pool at 48 h. *D,* immunoblot analysis of PKM2 and ADK proteins showing decreased expression when exposed to DHA for indicated times. Graphs are displayed as the mean ± SEM over three biological replicates. The statistical difference was analyzed by one-way ANOVA coupled to Dunnett's post hoc test or Student's *t* test and displayed as follows: ∗*p* < 0.05, ∗∗*p* < 0.01, and ∗∗∗*p* < 0.001. DHA, dihydroxyacetone; HU, hydroxyurea.

Replication stress arises from several factors, including nucleotide availability. RRM1 regulatory and RRM2 catalytic subunits of the RNR protein convert ribonucleotides to dNTPs. HU inhibits RNR. Because DHA replication stress was partially alleviated by removing the DHA, similar to HU, we reasoned that DHA may affect RNR. We first examined the transcript changes in RRM1 and RRM2 in BEAS-2B cells (Fig. S4*A*). DHA exposure did not alter the RRM1 transcript levels, but it significantly reduced the RRM2 transcript at 24 and 48 h post-exposure. We then examined the protein expression of RRM1 and RRM2 after exposure to 7.5 mM DHA for 1, 24, and 48 h (Fig. 5*A*). We observed significantly decreased RRM1 expression at 1 h of DHA exposure, which recovered at 24 and 48 h (Fig. 5*A*). We also observed significantly decreased RRM2 expression 24 h after DHA exposure (Fig. 5*A*). Compared with HU, DHA impacted RNR subunit expression but on different timescales and at different product levels (*i.e.*, transcript *versus* protein). The 48 h reduction of RRM2 is consistent with HU's mechanism, confirming that DHA impacts RNR over time but is less potent in its effects than HU (43).

Altered expression of RNR subunits suggests that alterations in nucleotide and deoxynucleotide pools may result from DHA exposure. In the cells, the nucleotide pool comprises mono-, di-, and tri-phosphorylated forms of nucleotides. The interconversion of nucleotides depends on the cell's energetics. However, only nucleotide diphosphates are converted to deoxynucleotide diphosphates for DNA biosynthetic pathways. We measured the nucleotide pool in DHA-treated BEAS-2B cells by performing LC–MS at 24 and 48 h. Quantification of AMP and ATP 24 h after DHA treatment showed no change; however, a slight increase in ADP was observed (Fig. 5*B*, *left*). Similarly, we observed a slight increase in ADP and a slight decrease in the ATP pool after 48 h DHA treatment (Fig. 5*B*, *right*). These changes were not statistically significant.

Because there were no substantial changes in AMP, ADP, or ATP in the DHA-treated cells, we measured the levels of dNTPs 24 and 48 h after DHA treatment. dNTP measurement was performed using a quantitative PCR–based method, where the incorporation of isolated nucleotides was detected on a detection site on the template DNA through a fluorescent dye (45). DHA treatment for 24 h did not affect dATP, dGTP, dCTP, and dTTP pools (Fig. 5*C*, *left*). However, after 48 h, a significant decrease in the dATP pool was observed, whereas dGTP, dCTP, and dTTP levels remained unchanged (Fig. 5*C*, *right*). Examination of proteins PKM2 and ADK involved in the conversion of phosphoenol pyruvate to pyruvate and phosphorylation of adenosine, respectively, showed no alteration in PKM2 protein but a decreased level of ADK (Fig. 5*D*). These results demonstrate that DHA exposure leads to specific depletion of the dATP pool, potentially through decreased ADK function.

### Adenine supplementation restores replication progression in DHA-treated cells

If DHA-induced replication stress originates from the depletion of the dATP pool, supplementing cells with ATP precursor adenine should rescue the replication stress. Adenine boosts the cellular levels of dATP through the nucleotide salvage pathway. Previous studies have shown that excess levels of nucleotides disrupt the relative nucleotide pool, leading to the inhibition of cell proliferation (46). Rescue of dATP imbalances could be achieved with adenine supplementation, though high levels of adenine may lead to cell death (46). Supplementation with 0.5 mM adenine only slightly impacted cell survival and boosted intracellular ATP levels in the BEAS-2B cells (Fig. S4, *B* and *C*). Adenine supplementation partially rescued DHA-induced cytotoxicity. Since DHA induces pleiotropic effects in cells, from genotoxicity to mitochondrial injury, we decided to examine if adenine could rescue the replication stalling induced by DHA despite its inability to fully rescue cytotoxicity.

**Figure 6:**
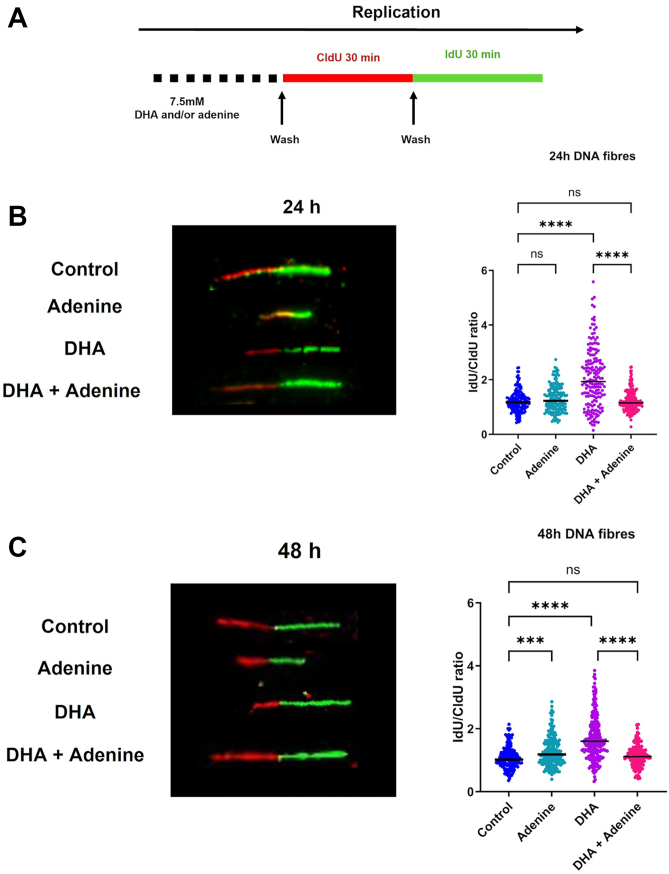
**Adenine supplementation in DHA-treated cells resolves replication stress.***A,* schematic of DHA and adenine treatment for fiber assay. *B,* representative fiber images and quantification of DNA fiber lengths in control, adenine (0.5 mM), DHA (7.5 mM), and adenine plus DHA-co-treated BEAS-2B cells at 24 h. *C,* representative fiber images and quantification of DNA fiber lengths 48 h after treatment. Fiber lengths represent arbitrary units measured from >150 fibers. Graphs are displayed as the mean ± SEM over three biological replicates. The statistical difference was analyzed by one-way ANOVA and Dunnett's post hoc test and displayed as follows: ∗*p* < 0.05, ∗∗*p* < 0.01, and ∗∗∗*p* < 0.001. DHA, dihydroxyacetone.

We again performed the DNA fiber assay with mock-treated control cells, cells treated with 0.5 mM adenine, 7.5 mM DHA, or co-dosed with adenine and DHA for 24 or 48 h (Fig. 6*A* and Fig. S4*D*). Cells were washed, and then CldU and IdU were added sequentially for 30 min. DNA fibers in 24 h adenine-supplemented cells have smaller CldU and IdU tracks with only a slight increase in the IdU–CldU ratio compared with the control (1.3 ± 0.03 *versus* 1.23 ± 0.03, *p* = 0.0007, Fig. 6*B*). DHA-treated and then washed cells showed a consistent increase in the IdU–CldU ratio of 2.0 ± 0.08 *versus* 1.23 ± 0.03 (*p* = 0.0001) (like Fig. 4*C*). Adenine supplementation enhanced the rescue of the replication stalling in the co-treated cells, with the ratio of IdU–CldU returning to 1.23 ± 0.02 (*p* = 0.33) compared with the control and DHA alone (*p* = 0.0001). After 48 h, we observed a similar trend with IdU–CldU ratio of 1.28 ± 0.03, 1.70 ± 0.05, and 1.15 ± 0.02 for adenine, DHA, and co-treated cells, respectively, compared with the control of 1.0 ± 0.02, indicating the reversal of DHA effects on CldU incorporation (Fig. 6*C*). Despite its inability to fully rescue the cytotoxicity of DHA, adenine supplementation did resolve the replication stalling and delayed progression induced by DHA.

### Adenine supplementation reduced micronuclei formation

**Figure 7:**
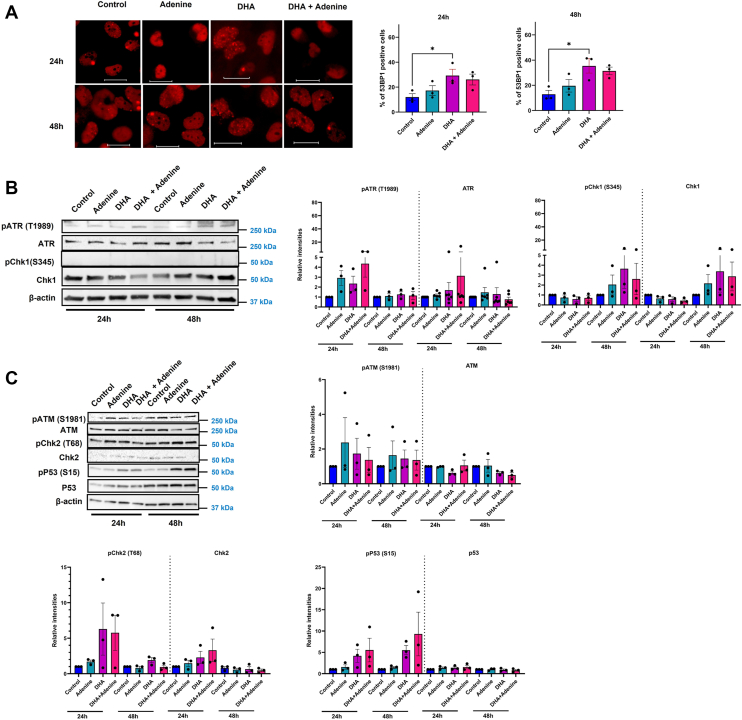
**Adenine supplementation alleviated DNA damage in DHA-treated cells.***A,* 53BP1 foci in control, adenine (0.5 mM), DHA (7.5 mM), and adenine plus DHA-co-treated BEAS-2B cells at 24 and 48 h after treatment. Cells with a minimum of six foci are considered as damage positive. The scale bar represents 12.5 μm. *B,* immunoblot analysis of ATR pathway proteins after DHA and DHA plus adenine co-exposure. *C,* immunoblot of ATM pathway proteins after DHA and DHA plus adenine co-exposure. Graphs are displayed as the mean ± SEM over three biological replicates. The statistical difference was analyzed by one-way ANOVA and Dunnett's post hoc test and displayed as follows: ∗*p* < 0.05, ∗∗*p* < 0.01, ∗∗∗*p* < 0.001, and ∗∗∗∗*p* < 0.0001. DHA, dihydroxyacetone.

We then examined the impact of adenine supplementation on the DNA damage and genomic instability induced by DHA. We first examined the effect of adenine on the induction of DSB breaks. Cells were dosed with adenine, DHA, or co-dosed with adenine and DHA, and 53BP1 foci were counted at 24 and 48 h after exposure. Adenine co-treatment with DHA did not significantly reduce the number of 53BP1 foci–positive cells (Fig. 7*A*).

We also evaluated the protein expression and activation of ATM- and ATR-mediated DNA damage signaling in adenine-supplemented cells. The addition of adenine to DHA-treated cells increased the activation of pATR T1989 at 24 h, but by 48 h, activation was lost (Fig. 7*B*). Similar trends were observed when pATR S428 levels were measured in adenine co-exposed cells (Fig. S5*A*). Co-exposure of DHA and adenine did not reduce pATR T1989 levels at 24 h but showed a small reduction at 48 h. Consistent with the lack of response observed earlier, we did not observe an alteration in pChk1 levels. Adenine supplementation also alleviated the increase in pATM and pCHK2 signaling and maintained CHK2 levels 48 h after exposure (Fig. 7*C*). Despite CHK2 being decreased, we did observe an increase in pP53 activation at 24 and 48 h. We examined the ratios of the phosphorylated proteins to their nonphosphorylated version and observed an increase in pATM/ATM and p-p53/p53 ratios even after adenine supplementation (Fig. S5*B*). We also examined if adenine supplementation could alter the presence of oxidative DNA lesions using oxRADD and observed decreased oxidative lesions when DHA and adenine were co-exposed (Fig. S5*C*).

**Figure 8:**
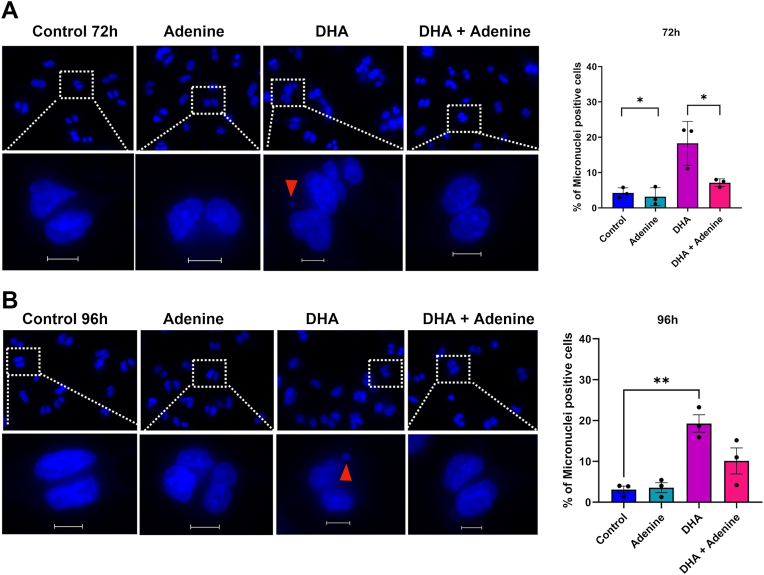
**Adenine supplementation only partially alleviated genomic instability induced by DHA exposure.***A,* immunofluorescent images and quantification of cytokinesis block micronuclei assay showing nuclei stained with Hoechst in control, adenine, DHA, and adenine plus DHA-co-treated BEAS-2B cells at 72 h. *B,* micronuclei images and quantification at 96 h. The scale bar represents 12.5 μM. Graphs are displayed as the mean ± SEM over three biological replicates. The statistical difference was analyzed by one-way ANOVA coupled with Dunnett's post hoc test or Student's *t* test and displayed as follows: ∗*p* < 0.05, ∗∗*p* < 0.01, ∗∗∗*p* < 0.001, and ∗∗∗∗*p* < 0.0001. DHA, dihydroxyacetone.

Finally, we tested whether adenine supplementation altered the formation of micronuclei. The number of micronuclei in adenine-supplemented cells was similar to the mock-treated control after 72 and 96 h after DHA treatment (Fig. 8, *A* and *B*). DHA exposure for 24 and 48 h followed by cytochalasin B showed a consistent increase in micronuclei. Co-treatment of DHA and adenine for 72 h significantly reduced the number of micronuclei. We also observed a reduction in micronuclei at 96 h after DHA and adenine co-treatment, though this reduction was not significant (Fig. 8*B*). These assays demonstrate that adenine supplementation alleviated replication stress and reduced micronuclei formation in BEAS-2B cells after DHA exposure. However, it only partially rescues the cells from micronuclei formation. These data demonstrate that adenine supplementation partially alleviates DHA-induced genomic instability.

## Discussion

Our recent examinations of DHA's genotoxicity in systemic liver, lung, and cardiac models using low millimolar doses (5–15 mM) have demonstrated that DHA is cytotoxic, a metabolic disruptor, and causes cell cycle arrest dependent on cell type (5, 7, 8, 9). Previous studies in the skin used 5 to 50 mM doses to establish the cytotoxicity of DHA and its induction of AGEs (4, 10). These dose ranges may be appropriate for skin applications where the sensitivity of dermal fibroblasts and keratinocytes appears less sensitive to DHA effects (4, 6, 10). Our previous studies established that kidney, liver, lung, and cardiac models are more sensitive to DHA's effects than skin (5, 7, 8, 9). However, it is less clear what doses are relevant for e-cigarette exposures or systemic exposures.

During e-cigarette use, DHA is produced at 0.16 to 4.16 μg per 100 ml puff (2). E-cigarette puff volumes are reported at 96.81 to 133.92 ml per puff for cigarette-like devices and at 331.2 to 519.6 ml for tank devices (47, 48). Most studies suggest an average of 10 puffs per session, with vapers engaging in as many as 24 sessions per day (49, 50). Therefore, a single inhalation could be as high as 1 μM DHA, with daily exposure because of vaping ranging from 200 μM to 1 mM DHA (2, 51). While these are just estimates, low millimolar doses of DHA are relevant for studying its cellular effects for e-cigarette and sunless tanning exposures.

At these doses, we have demonstrated that DHA induces oxidative and crosslink-type DNA lesions and promotes chromosomal instability (Figs. 1 and 4) (5). In our previous work, AC16 cells did not experience cell cycle arrest but did show DNA damage, highlighting the cell-specific effects of DHA (5). None of the systemic cells, AC16, A549, BEAS-2B, HEK293T, or HepG3, showed phosphorylated H2AX (γH2AX), a DNA damage signal, or ROS generation (5). However, they did show weak 53BP1 foci formation. In particular, BEAS-2B cells showed a significant accumulation of 53BP1 foci at their IC_90_ doses (12.5 mM), suggesting replication stress or ongoing DSB formation and impaired repair.

Given these findings, we examined replication stress in the BEAS-2B cells at a 7.5 mM dose where cell death would not dominate. We started by examining the presence of DNA damage and associated ROS. Similar to previous work, we did not observe an increase in total cellular ROS after DHA exposure for 24 and 48 h (Fig. 1*A*). The DCFDA-based fluorescent assay is a general ROS sensor, so we cannot discount that other ROS or reactive nitrogen species are generated (52, 53). We confirmed that oxidative stress is present after DHA exposure by confirming oxidative DNA lesions recognized by FPG and endo VIII are present along with an increase in oxidized proteins (Fig. 1, *B* and *D*). These results confirm that other reactive species are induced by DHA, possibly lipid peroxidation or superoxide. Protein carbonylation can also occur from metal-catalyzed oxidation and α-amidation/oxidation of glutamine residues or through indirect sources of oxidation, such as lipid peroxidation or formation of AGEs. Carbonylation of protein could be a significant source of enzyme dysfunction, protein aggregation, and chromatin disruption (54). Our previous study did not measure significant increases in AGEs, so lipid peroxidation or other reactive species, like aldehydes, may be the source of oxidative stress after DHA exposure.

We also observed an increase in crosslink and uracil lesions after DHA exposure. T4PDG recognizes the crosslink-type lesions induced by DHA. These crosslink-type lesions appear to be similar to CPD lesions, given that they are recognized by the anti-CPD antibody in our slot blot (Fig. S1*C*). However, more work is needed to understand the structure of these lesions. Pyrimidine dimers present physical obstacles to transcription and replication machinery and hinder DNA metabolism (55, 56). Increased uracil content in the genome is also associated with replication stress and genomic instability (57).

Given the complex mixture of DHA-induced DNA damage and the lesions' ability to induce replication stress, we examined the activation of DNA damage response after DHA exposure. Using multiple markers, we confirmed that DSB repair signaling was significantly elevated after DHA exposure, measured through increased 53BP1 foci, pATM/ATM ratio, and pChk2 and pP53 protein levels. The only hallmark of SSB signaling observed was elevated pATR T1989 levels over the mock-treated control, but the S428 phosphorylation site was not elevated (Figs. 2*B* and S3*C*). Surprisingly, ATR activation in DHA-exposed cells lacked a downstream activation of Chk1 despite the presence of oxidative lesions, indicating a complicated interaction of DHA with ATR pathway (Figs. 2*B* and S3*D*). The absence of canonical ATR response through pChk1 activation indicates either the absence of a complete complex assembly at the damage site, which includes recruitment of claspin and TopBP1, or ATR is required for the replication fork protection and stability rather than global checkpoint activation in DHA-exposed cells. In either scenario, ATR activation under DHA exposure requires further investigation.

While DSB signaling was elevated, we did not observe cell cycle arrest with 7.5 mM dose of DHA (Fig. 3). Our data showed a slight increase in S-phase cells, corresponding with the accumulation of S-phase CDK2 (Fig. 3*B*). The percentage of cells arrested in the S phase in the presence of a 7.5 mM dose of DHA (control 6.77 ± 1.94 *versus* 18.30 ± 3.10 at 48 h) are similar to the percentage of cells arrested with a cytotoxic dose of DHA (control 16.3 ± 1.94 *versus* DHA 23.70 ± 1.90) (5). This suggests that a small percentage of S phase-arrested cells survive, or DNA damage response recovered cells from the arrest. These outcomes may result in the chromosomal aberrations observed at the IC_90_ dose (5). We also observed decreased levels of replication proteins BLM and WRN (Fig. 3*C*). WRN and BLM are members of the RecQ family of helicases and are involved in recovery from replication stress in the cells during S-phase (44). BLM is involved during the early stages of the S-phase when the replication fork is progressing. In DHA-exposed cells, BLM is significantly decreased only at 48 h of treatment, indicating that recovery from replication stress is possible during the early hours of treatment. WRN is involved in the repair of replication-associated damage and telomere maintenance during the later stages of the cell cycle. We observed a robust decline in WRN levels, which may impact postreplication DNA repair and telomere maintenance in the cells. Changes in WRN levels may influence the chromosomal instability we observed after DHA exposures.

We confirmed chromosomal instability by observing increased micronuclei formation 72 and 96 h post-DHA exposure. The increase in the percentage of micronuclei between 72 and 96 h of DHA exposure suggests that longer exposure time resulted in increased genomic instability in the cells (Fig. 4*D*).

Oxidative stress in the form of protein carbonylation and oxidative lesions may be related to continued replication stress induced by the presence of DHA. Therefore, we specifically confirmed the presence of replication stress after DHA exposure using a DNA fiber assay. We first conducted the fiber assay in the presence of DHA and noted significant defects in the incorporation of CldU and IdU but not fork collapse or stalling (Fig. 4*B*). We interpreted these results as a significant slowing of the replication fork in the presence of DHA. We then repeated the fiber assay, removing DHA before adding the thymidine analogs. Interestingly, the removal of DHA alleviated the slow fork progression but only after 30 min in DHA-free media (Fig. 4*C*). These changes were similar to the reversible effects of HU exposure for short durations, and like HU, we observed changes in the expression of RRM1 and RRM2 after DHA exposure. DHA, unlike HU, kept RRM1 reduced 48 h after exposure, suggesting RRM1 may be inhibited or damaged in the presence of DHA, which is unique compared with HU (Fig. 5*A*).

When we examined the nucleotide pools, we observed fluctuations in the nucleotide levels but only a significant decrease in dATP. A decrease in a single nucleotide is unexpected given the equilibrium reactions employed by the cells, but this decrease coincided with a decrease in ADK, which converts adenosine to ATP (Fig. 5, *C* and *D*). Changes in ADK are unique to DHA exposure and suggest protein damage may result in the loss of dATP.

With the specific loss of dATP, we tested whether adenine supplementation could rescue DHA-mediated cell effects. While adenine rescued the replication stress observed after DHA exposure, it could not completely rescue cell viability or micronuclei formation induced by DHA (Figs. 6,8 and S4*B*). In addition, we saw a persistence of 53BP1 foci and DNA damage signaling even after adenine supplementation (Fig. 7*A*). The inability of adenine to resolve 53BP1 foci and eliminate DNA damage signaling indicates there are still persistent DNA lesions or strand breaks induced by DHA, which may not be associated with the replication fork. We propose a model in which DHA triggers the formation of complex DNA lesions, as indicated by oxRADD and T4PDG, and induces oxidative damage to replication-associated proteins, including RRM1 subunits. DHA alters RNR levels partly through transcriptional regulation (Fig. S4*B*). This mixture of damage leads to slowed replication fork progression, early activation of DNA damage response, and a modest disruption of dNTP pool by 24 h before any significant changes in dATP pool. By 48 h, continued stress, likely from unrepaired crosslink lesions coinciding with the onset of metabolic stress, leads to dATP depletion, which worsens replication stress and genomic instability. This highlights a critical link between metabolic stress, nucleotide homeostasis, and DNA replication fidelity in the DHA-exposed cells. Adenine appears to promote the repair of oxidative lesions as measured by the oxRADD assay (Fig. S5*C*). In addition, it alleviates the replication stress through enrichment of the purine pool, thereby facilitating uninterrupted replication fork progression. However, adenine might not be reversing crosslinked DNA or DHA-induced protein damage by 48 h, which likely leads to persistent DDR and micronuclei formation.

These observations suggest that DHA induces replication stress through a multifactorial mechanism involving DNA damage, oxidative protein damage, and metabolic changes in nucleotide pools. While replication stress because of metabolic imbalance is reversible, the DNA damage resulting from fork collapse, secondary structure formation, or lesions is not readily resolved by adenine supplementation alone. Further research is required to understand the factors that drive micronuclei formation after DHA exposure. It may be the persistent strand breaks associated with 53BP1, or the slowing of DNA repair processes because of the continued presence of DHA, or unknown mitotic defects induced by DHA's fluctuations of NAD(P)H or ATP pools (7, 8, 9). We previously observed a significant elevation in chromosomal aberrations, including breaks and ploidy changes, at an IC_90_ dose (12.5 mM). Mitotic defects were not previously reported in skin studies; however, Striz *et al.* (4) noted an increase in G2/M checkpoint proteins, including Aurora A, using microarray analysis after 5 mM DHA exposure to keratinocytes.

Together, these results demonstrate that the depletion of dATP by DHA slows replication fork progression, contributing to DHA's genotoxicity. The exact mechanism by which dATP is depleted by DHA exposure is still unclear, but it does not result from a loss in ATP, which increases after DHA exposure in BEAS-2B cells (Figs. 5*B* and S4*C*). Protein loss by ADK is the most likely explanation, but the lack of impact on the other adenine nucleotides needs further investigation. Increased metabolic flux through glycolysis may create an increase in ATP, but the inability to convert this to dATP still needs explanation. We also determined that chromosomal instability, another genotoxic effect of DHA, is independent of the replication fork changes and cannot be completely rescued by adenine supplementation. Further research is necessary to understand if DHA induces mitotic defects or inhibits mitotic processes. While the mechanisms of action associated with DHA are still being determined, we have demonstrated that DHA is a complex genotoxin that induces replication stress and genomic instability in BEAS-2B cells at 7.5 mM DHA doses.

## Experimental procedures

### Chemicals

DHA (catalog no.: PHR 1430), CldU (C6891), IdU (I7125), and bromodeoxyuridine (BrdU) (5002) were purchased from Sigma–Aldrich. Cytochalasin B (228090010) was purchased from Thermo Scientific.

### Cell culture

The human bronchial epithelial cell line (BEAS-2B, CRL-3588) was purchased from American Type Culture Collection every 6 months and cultured or no more than 10 passages for the presented experiments. The BEAS-2B cells were cultured in RPMI1640 supplemented with 10% fetal bovine serum (Corning). The cells were screened monthly for mycoplasma contamination using the Lonza Mycoalert kit) monthly.

### ROS measurements

Total intracellular ROS was measured in BEAS-2B cells using CM-H_2_DCFDA (C6827; Thermo Fisher). Five thousand cells per well in a clear bottom, black 96-well plate and left to grow overnight (ON) in a 5% CO_2_ incubator at 37 °C. After attachment, cells were dosed with 7.5 mM DHA (IC_50_) for 24 or 48 h (5). As a positive control, cells were dosed with 250 μM tert-butyl hydrogen peroxide (AAA13926AE; Fisher Scientific) for 1 h. After exposure, 10 μM of CM-H_2_DCFDA was added to each well and incubated for 30 min at 37 °C. Once the incubation period was complete, fluorescence was measured at 493/522 nm using the BioTek Synergy HR microplate reader (Agilent Technologies). After reading the total ROS measurement, cells were stained using Hoechst staining solution at 1:1000 (62249; Thermo Fisher) and incubated for 15 min at room temperature (∼22 °C). Fluorescence was read again at 366/460 nm to normalize fluorescence to the cell count in the well. Values were graphed relative to control using GraphPad Prism and displayed as mean and SEM values of three biological replicates. Significance was calculated using the one-way ANOVA with Dunnett's post hoc test.

### Repair assisted damage detection

DNA damage was evaluated using RADD, which detects DNA lesions using a specific cocktail of DNA repair enzymes. These enzymes remove the DNA lesion, and then the gap or strand break is tagged using a digoxigenin-labeled dUTP inserted with Klenow exo-, which lacks proofreading (41, 58). We used specific lesion cocktails to evaluate oxidative lesions, crosslinks, alkylation, or uracils within the genomic DNA. For oxidative lesions only (oxRADD), FPG (M0240; New England BioLabs [NEB]), endo IV (M0304; NEB), and endo VIII (M0299; NEB) were used. For crosslinks (T4PDG, M0308; NEB), only T4 pyrimidine dimer glycosylase (T4PDG) and endo IV were used. For uracil detection, uracil DNA glycosylase (UDG, M0280; NEB) and endo IV were added. We assessed DNA lesions within the BEAS-2B cells. The cells were plated in 8-well chambers at a density of 5000 cells and left to attach ON in a 5% CO_2_ incubator at 37 °C. The next day, cells were mock treated, dosed with 7.5 mM DHA for 24 and 48 h, or with 25 μM MG (M0252; Sigma–Aldrich) as a positive control. After the exposure, cells were fixed using 3.7% formaldehyde (BP531; Fisher Scientific) for 10 min and washed three times with PBS (SH30028F2; Fisher Scientific) following fixation. Biotium permeabilization buffer (22016; Fremont) with 0.05% Triton-X permeabilized the cells for 10 min at 37 °C. The cells were then washed three times with 1X PBS.

Chambers were then incubated with lesion removal cocktails described above and resuspended in 1X ThermoPol buffer + bovine serum albumin (BSA) (B9004; NEB) for 1 h at 37 °C in a hybridization FISH oven. Once the incubation period has finished, the gap-filling mixture of Klenow exo- (FEREP0422; Fisher Scientific) and digoxigenin-labeled dUTP (11093088910; Sigma) was directly added to the chambers and placed again within the oven at 37 °C for an additional 1 h. The chambers were washed three times with 1X PBS and blocked with 2% BSA (001-000-162; Jackson Immunological) in PBS for 30 min. After blocking, chambers were incubated with primary antibody antidigoxigenin (1:250 dilutin, ab420; Abcam) or anti-mouse IgG1 isotype control (1:625 dilution, 5415; Cell Signaling) for 1 h at room temperature. IgG1 was used as a negative control in these cells. Once the primary antibody had finished incubation, the cells were washed three times with 1X PBS and incubated with secondary antibody, anti-mouse AlexaFluor 546 (1:400 dilution, A11003; Thermo Fisher) for 1 h at room temperature. The cells were then incubated with Hoechst solution (1:800 dilution) for 15 min at room temperature and washed with 1X PBS three times.

Cells were then imaged using the Keyence microscope (BZ-X800; Keyence) with the 20X objective (numerical aperture [NA] = 0.75). At least 30 cells of each condition for each biological replicate were taken over four separate fields. For analysis, the Nikon Elements software created an area of interest (region of interest [ROI]) around the nucleus, and the total intensity for the RADD channel within the nucleus was recorded for at least 300 cells. The fluorescent intensity for all the nuclei was graphed in GraphPad Prism, and significance was calculated using a one-way ANOVA with Dunnett's post hoc test for each DNA adduct category.

### Protein carbonylation

Protein carbonylation was detected using OxyBlot Protein Oxidation Detection Kit (S7150; Sigma–Aldrich). Briefly, BEAS-2B cells were plated at 0.5 × 10^6^ in 6-cm dishes and left in a 5% CO_2_ incubator at 37 °C to attach for ON. After attachment, cells were mock treated with media or dosed with 7.5 mM DHA for 24 and 48 h. After the exposure period, the cell medium was collected in a 15 ml conical tube, and the cells were detached using cell scrapers and resuspended in the collected cell medium. Cells were pelleted down at 2000 rpm for 5 min. Supernatants were aspirated, the cell pellets were lysed using radioimmunoprecipitation assay lysis buffer, and protein quantification was done using Bradford reagent (23238; Thermo Fisher). Protein (5 μg) was used to detect protein carbonylation using the user manual provided with the kit. Detected bands of three biological replicates were quantified using the ImageJ software (National Institute of Health and the Laboratory for Optical and Computational Instrumentation, University of Wisconsin) by taking total intensities of each lane and calculated relative to the loading control β-actin and then the untreated cells. Values were plotted using GraphPad Prism and displayed as the mean and SEM values. Statistical analysis was performed on values using the one-way ANOVA with Dunnett's post hoc test.

### Immunofluorescence

Cells were plated in fluorodishes (50823005; Thermo Fisher) at a density of 70,000 cells per well. The cells were allowed to adhere ON in a 5% CO_2_ incubator at 37 °C. After attachment, cells were dosed with 7.5 mM DHA for 24 and 48 h. After exposure, cells were fixed with 3.7% formaldehyde in PBS for 10 min at room temperature and washed three times with 1X PBS. Then, cells were permeabilized using Biotium permeabilization buffer for 10 min at room temperature and washed three times with PBS. Cells were blocked for 30 min at room temperature using 2% BSA in PBS. The cells were then incubated with the corresponding primary antibody, RPA32 (1:1000 dilution, ab2175; Abcam) and 53BP1 (1:750 dilution, NB100-304; Novus Biologicals) for 1 h at room temperature. After incubation, cells were washed three times with 1X PBS to remove any excess antibodies; then, they were incubated for 1 h with anti-rabbit Alexa Fluor 546 (1:400 dilution, A11010; Thermo Fisher) for 1 h at room temperature. Nuclear staining was performed using Hoechst solution (1:1000 dilution) for 10 min before the end of the incubation period. Then, cells were washed three times with PBS and imaged. Imaging was conducted with the all-in-one fluorescence Keyence microscope using the 60X objective (NA = 1.4). A minimum of 100 cells were imaged for each condition over three biological replicates. The Nikon Elements software was used to define the ROI for the nucleus, and the mean fluorescent intensity was calculated with this ROI for each condition. GraphPad Prism was used to display the values ± SEM over the replicates. Significance was calculated using one-way ANOVA and Dunnett's post hoc test.

### Immunoblotting

Immunoblotting was performed as previously described [20]. BEAS-2B cells were plated at 0.5 × 10^6^ in 6-cm dishes and left in a 5% CO_2_ incubator at 37 °C to attach for ON. After attachment, cells were mock treated with media or dosed with 7.5 mM DHA for 1, 24, and 48 h, 4 mM HU (151680050; Fisher Scientific) for 5 h, or 250 nM CPT (C1495; Fisher Scientific) as a positive control for 24 h. After the exposure period, the cell medium was collected in a 15 ml conical tube, and the cells were detached using cell scrapers and resuspended in the collected cell medium. Cells were pelleted down at 2000 rpm for 5 min. Supernatants were aspirated, and the cell pellets were stored at −80 °C ON.

The next day, cells were lysed, and protein quantification was performed using a Bradford assay. Protein (20–40 μg) was loaded in 4% to 20% Mini-PROTEAN Precast Gels (Bio-Rad) and ran at 130 V for 1 h. The gels were transferred using a nitrocellulose membrane and blocked for 1 h using 5% skim milk in 1X Tris-buffered saline (TBS, ICN08W00020; Fisher Scientific) with 0.1% Tween-20 (TBST, AAJ20605AP; Fisher Scientific). The membranes were then incubated ON with corresponding primary antibodies (Table 1). Abcam antibodies are validated by knockout by the manufacturer, whereas Cell Signaling Technology uses a six-part validation strategy to confirm antibody activity as recommended (59). The next day, membranes were washed three times with TBST for 5 min each and incubated with corresponding horseradish peroxidase–conjugated secondary antibodies (7076 or 7074; Cell Signaling Technology) for 1 h at room temperature. After incubation, membranes were washed three times with TBST, and chemiluminescence (electrochemiluminescence, K-12043-D20; Advansta) was used to image the membranes using the Bio-Rad ChemiDoc imaging system. Detected bands of three biological replicates were quantified using the ImageJ software and calculated relative to the loading control and then the untreated cells. Values were plotted using GraphPad Prism and displayed as the mean and ± SEM values. Statistical analysis was performed on values using the one-way ANOVA with Dunnett's post hoc test.

**Table 1:** Antibodies used for immunoblotting experiments

Antibody	Dilution	Source
CDK2 (#18048)	1:1000	Cell Signaling Technology
ATM (#2873)	1:1000	Cell Signaling Technology
ATR (#2790)	1:1000	Cell Signaling Technology
pATM-S1981 (#5883)	1:500	Cell Signaling Technology
pATR-S1989 (#30632)	1:1000	Cell Signaling Technology
pATR-S428 (#2853)	1:500	Cell Signaling Technology
CHK1 (#2360)	1:1000	Cell Signaling Technology
CHK2 (#6334)	1:1000	Cell Signaling Technology
pCHK1-S345 (#2348)	1:500	Cell Signaling Technology
pCHK2-T68 (#2197)	1:500	Cell Signaling Technology
pP53-S15 (#9286)	1:1000	Cell Signaling Technology
Mouse Anti-IdU (#347580)	1:50	BD
Rat Anti-bromodeoxyuridine (#ab6326)	1:50	Abcam
RRM1 (#3388)	1:1000	Cell Signaling Technology
BLM (#2742)	1:1000	Cell Signaling Technology
WRN (#4666)	1:1000	Cell Signaling Technology
PKM2 (#3198)	1:1000	Cell Signaling Technology
ADK (#93994)	1:1000	Cell Signaling Technology
pRPA32-S33 (#10148)	1:1000	Cell Signaling Technology
RPA32 (#2208)	1:3000	Cell Signaling Technology
RPA32 (#ab2175) (IF)	1:1000	Abcam
53BP1 (NBP100-304)	1:500	Novus Biological
β-actin (#AM4302)	1:5000	Invitrogen

### Cell cycle analysis

To evaluate cell cycle arrest, BEAS-2B cells were plated at 1 × 10^6^ and incubated ON at 37 °C in a 5% CO_2_ incubator for attachment. Cells were then mock treated as a control or exposed to 7.5 mM values of DHA for 24 and 48 h. After exposure, cells were collected and prepped for cell cycle analysis through flow cytometry, as described (5). Briefly, the medium was collected from plates, and cells were washed using 1X PBS once. The cells were detached using 0.25% trypsin–EDTA and resuspended in the collected medium. The samples were centrifuged; the pellet was washed with PBS and centrifuged again. The cells were counted, and 10^6^ cells were aliquoted into a new tube and centrifuged. The pellet was resuspended in ice-cold PBS, and the cells were fixed using 70% ethanol and left ON at 4 °C. The next day, samples were centrifuged, and the pellet was washed once with 1X PBS and centrifuged again. The samples were resuspended in RNase A, incubated for 10 min at 37 °C, and then stained with propidium iodide (P3566; Thermo Fisher) for 15 min at room temperature. The samples were run for cell cycle analysis using the BD FACS Symphony (BD Biosciences) and analyzed using the FlowJo software (BD Biosciences). The graphs displayed are a representative run, and the values calculated are the average percentage of each cycle ±SEM of the three biological replicates.

### DNA fiber assay

Briefly, cells were seeded at 0.6 × 10^6^ in 6 cm dishes and left in a 5% CO_2_ incubator at 37 °C to attach for ON. After attachment, cells were mock treated with media, dosed with 7.5 mM DHA for 24 and 48 h, or dosed with HU at 4 mM for 5 h. At the end of the treatment, cells were pulse-labeled with 100 μM CldU and 100 μM IdU for 30 min. The cells were then harvested and resuspended in 50 μl of chilled 1X PBS. The cell suspensions (3 μl) were placed on glass slides (50-949-515; Superfrost) and allowed to air dry. The cell suspension was then mixed with 8 μl of lysis buffer (0.5% SDS 97602-964, VWR; 200 mM Tris–HCl [pH 7.4] [T1503; Sigma], 50 mM EDTA [AM9260G; Thermo Fisher]) and incubated for 2 min. The slides were inclined at an angle between two in a 3:1 solution of methanol–acetic acid (Fisher Scientific) for 10 min, followed by denaturation using 2.5 N HCl for 80 min. After three to five rinses in 1X PBS, the slides were incubated with a blocking buffer (2% BSA and 0.01% Tween-20 in PBS) for 15 min. This was followed by incubation with rat anti-bromodeoxyuridine (CldU) antibody (Table 1) for 90 min in a humidified chamber at room temperature. The slides were washed once with 0.1% Tween-20 and fixed in 4% formaldehyde solution for 15 min. After five PBS washes, the slides were incubated with an anti-rat FITC secondary antibody (Table 1). After incubation for 1 h at room temperature, the slides were washed twice with 0.1% Tween-20 and incubated ON with mouse anti-IdU antibody (Table 1) at 4 °C. The slides were washed twice with PBS and incubated with an anti-mouse TRITC antibody (Table 1). After two washes with 1X PBS containing 0.1% Tween-20, slides were mounted with coverslips using 70% glycerol in 1X PBS. Imaging was conducted with the Keyence microscope using the 60X objective (NA = 1.4). A minimum of 200 fibers were imaged for each condition over three biological replicates. The ImageJ software was used to measure the lengths of each label. GraphPad Prism was used to display the values ± SEM over the replicates. Significance was calculated using one-way ANOVA and Dunnett's post hoc test.

### Cytokinesis-block micronuclei assay

BEAS-2B cells were plated at 1 × 10^6^ cells in fluorodishes and incubated ON at 37 °C in a 5% CO_2_ incubator for attachment. Cells were then mock treated or exposed to 7.5 mM DHA for 24 and 48 h. At the end of the treatment, 3 μg/ml cytochalasin B was spiked into each plate and incubated for 48 h. After treatment, cells were fixed with 3.7% formaldehyde (Thermo Fisher) in PBS for 10 min at room temperature and washed three times with 1X PBS. Nuclear staining was performed using Hoechst solution (1:1000 dilution) for 10 min followed by three washes with 1X PBS. Imaging was conducted with the all-in-one fluorescence Keyence microscope using the 60X objective (NA = 1.4). GraphPad Prism was used to display the values ± SEM over the replicates. Significance was calculated using a Student's *t* test.

### Nucleotide pool measurement

We used the protocol outlined in (45) to measure the dNTP pool. For dNTP extraction, cells were plated in 6-well plates at 2 × 10^5^ cells/well and allowed to adhere and grow for 16 to 18 h. Cells were either mock treated or treated with 7.5 mM DHA and incubated for 24 or 48 h. Cells were washed twice with PBS and detached using 0.25% trypsin–EDTA (Thermo Fisher). The cell suspension (10 μl) was used for counting, and the rest of the cell samples were centrifuged at 3000 rpm for 5 min at 4 °C and resuspended in 500 μl of 60% ice-cold methanol by vortexing. Samples were incubated for 3 min at 95 °C to quench residual enzymatic activity and facilitate extraction. The samples were centrifuged at 18,500*g* for 6 min at 4 °C to remove cell debris. The supernatants were passed through a pre-equilibrated Amicon Ultra-0.5-ml centrifugal filter Unit (UFC500324, Sigma–Aldrich) to remove macromolecules according to the manufacturer's instructions. Then, methanol and hydrophobic metabolites were removed by washing twice with 1.4 ml diethyl ether. Residual diethyl ether was removed using a Speed vacuum concentrator (Biotron) for 15 min, and the resultant pellet was resuspended in 100 μl nuclease-free water/1 × 10^6^ cells and assayed immediately or stored at −80 °C until use.

Q5 DNA polymerase and EvaGreen-based assay for dNTPs using 197-nt templates. For 2× master mix, the final concentrations of components were the following: 2× Q5 reaction buffer, 0.5 μM primer, and 0.5 μM 197-nt template, 0.1 mM dNTPs (except for target dNTP) and 2.5 μM EvaGreen (Biotium). Q5 DNA polymerase (M0493; NEB) was then added to reaction mixtures as follows: final concentration of 20 U/ml for dATP and dTTP and 10 U/ml for dGTP and dCTP detection. Five μl of each 2× master mix was pipetted into Hard-Shell 96-Well PCR Plates (Bio-Rad) according to dNTP species. Then, 5 μl of sample was added to each well for dNTP quantification by real-time PCR. For initial optimization runs, the thermal cycler (CFX96; Bio-Rad) was programmed to heat the plate to 98 °C (10 s), followed by cooling to the final reaction temperature (67 °C). The baseline fluorescence was immediately read after reaching the target temperature. Thereafter, the fluorescence (SYBR Green/FAM channel of the instrument) was recorded typically once every 5 min for a total of 75 min. Data were normalized to the volume of estimated samples. The baseline-corrected fluorescence intensities from three biological replicates were plotted in GraphPad Prism and displayed as mean ± SEM. Significance was calculated using the Student's *t* test.

### Mass spectrometry sample preparation

For mass spectrometry, BEAS-2B cells were plated at 2 to 3 × 10^6^ cells in 15 cm dishes and incubated ON at 37 °C in a 5% CO_2_ incubator for attachment. Cells were then mock treated as a control or exposed to 7.5 mM of DHA for 24 and 48 h. At the end of the treatment, dishes were transferred to the cold room, and media were aspirated. Plates were washed twice with cold 1X PBS, and after the wash, liquid nitrogen was directly added to the dishes and allowed to boil off. When the dishes started to thaw, the dish surface was scraped using a cell scraper, and the cell slush was transferred to 1.5 ml tubes. Once the cell slush is completely thawed, 10 μl is aliquoted for protein quantification, and the remaining samples were frozen at −80 °C until sent to the metabolic core at Perelman School of Medicine, University of Pennsylvania.

### LC–MS quantitation of nucleotides

Frozen cell lysates were homogenized in ice-cold 80% methanol. Aliquots (∼100 μl) of homogenate were spiked with 10 μl of isotopically labeled nucleotides as internal standards, extracted with 400 μl of methanol, and centrifuged at 18,000*g* for 5 min at 4 °C. Then, 400 μl of the supernatant was dried under nitrogen at 45 °C and reconstituted in HPLC solvents for LC–MS. Calibration solutions (10 μl) and internal standards (10 μl) were spiked into 90 μl of 80% methanol and similarly prepared. Separation and quantification of nucleotides was achieved with a 9 min linear gradient (95–46% acetonitrile with 8.6 min re-equilibration, 0.6 ml/min, 40 °C, 1 μl injection) on a Waters Atlantis Premier HILIC-z column and multiple reaction monitoring of calibration solutions and study samples on an Agilent 1290 Infinity UHPLC/6495 triple quadrupole mass spectrometer. Raw data were processed using Mass Hunter quantitative analysis software (Agilent). Calibration curves (*R*^2^ = 0.99 or greater) were either fitted with a linear or a quadratic curve with a 1/X or 1/X^2^ weighting.

### Cell viability assay

Cell viability was determined using a cell count assay as previously described [20]. BEAS-2B cells were plated at 5000 cells per well in a 12-well and left ON in a 5% CO_2_ incubator at 37 °C. The next day, cells were either mock treated or exposed to 7.5 mM DHA, 0.5 mM adenine (A2786; Sigma–Aldrich), or co-treated with DHA and adenine. The treated cells were grown for 5 to 7 days. For counting, cells were harvested using 0.25% trypsin–EDTA and then resuspended in 1X PBS. Cells were counted using the Bio-Rad TC automated cell counter (Bio-Rad). The results are graphed as the mean survival ± SEM for three biological replicates. GraphPad Prism was used to plot the data and calculate the significance using one-way ANOVA with a Dunnett's post hoc test.

## Data availability

This article contains supporting information. All relevant data are within the article and its supporting information files. Blot images can be found at https://doi.org/10.6084/m9.figshare.30175576.

## Supporting information

This article contains supporting information.

## Conflict of interest

The authors declare that they have no conflicts of interest with the contents of this article.
