# Supplementary material for: Dihydroxyacetone decreases the dATP pool, inducing replication stress and genomic instability in BEAS-2B cells
Source: J Biol Chem. 2025 Oct 30;301(12):110876. doi: 10.1016/j.jbc.2025.110876 (PMC12682131; doi:10.1016/j.jbc.2025.110876)
Supplement: Supporting Information [file mmc1.pdf]

# Supporting Information

**Dihydroxyacetone decreases the dATP pool, inducing replication stress and genomic instability in BEAS-2B cells**

Saddam Hussain[1], Nayonika Mukherjee[1], and Natalie R. Gassman[1,*]

## List of Supporting Figures

**Supporting Figure 1.** Quantification of DHA induced lesions in BEAS-2B cells

**Supporting Figure 2.** DNA repair pathways are intact in DHA exposed BEAS-2B cells.

**Supporting Figure 3.** Examination of DNA damage response in BEAS-2B cells.

**Supporting Figure 4**. Impact of DHA and adenine supplementation on ATP levels and BEAS-2B cell survival.

**Supporting Figure 5.** Impact of DHA treatment and adenine supplementation on DNA damage response and oxidative lesions in BEAS-2B cells.

## Supporting Methods

To develop pRPA32 blots, 10X cell lysis buffer (#9803) from CST is recommended. Briefly cells were plated in 10cm dishes and dosed as mentioned in the material and methods section. At the end of the treatment cells were harvested as recommended in cell lysis buffer method. At the cell sonication step, cell lysate was sonicated for 12 cycles of 5s on and 5s off for total of 2 minutes. Membrane blocking and antibody dilutions were made in 5% BSA in 1X PBS.

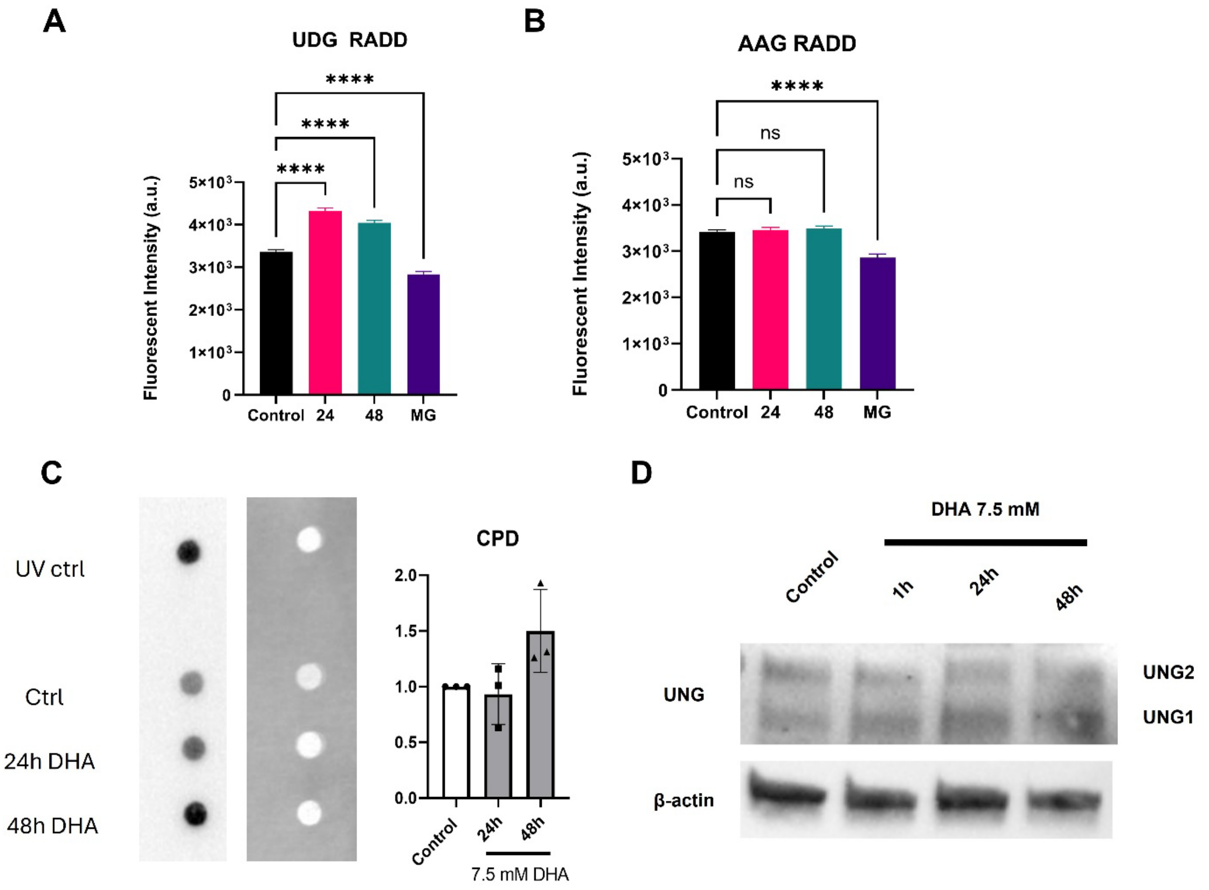


**Supporting Figure 1.** Quantification of DHA induced lesions in BEAS-2B cells (A) Measurement of uracil and (B) alkylation lesions after exposure to 7.5 mM DHA for 24 and 48h. 25 µM methylglyoxal was taken as a positive control. (C) Dot blot of DNA-protein crosslink in DHA exposed BEAS-2B cells at 24 and 48 h. Exposure to UV light was taken as a positive control. (D) Immunoblot of UNG protein in DHA exposed BEAS-2B cells at 1, 24 and 48 h. Graphs are displayed as the mean ± SEM over three biological replicates. The statistical difference was analyzed by one-way ANOVA coupled with Dunnett's post hoc test and displayed as follows: *p<0.05 and ****p<0.0001.

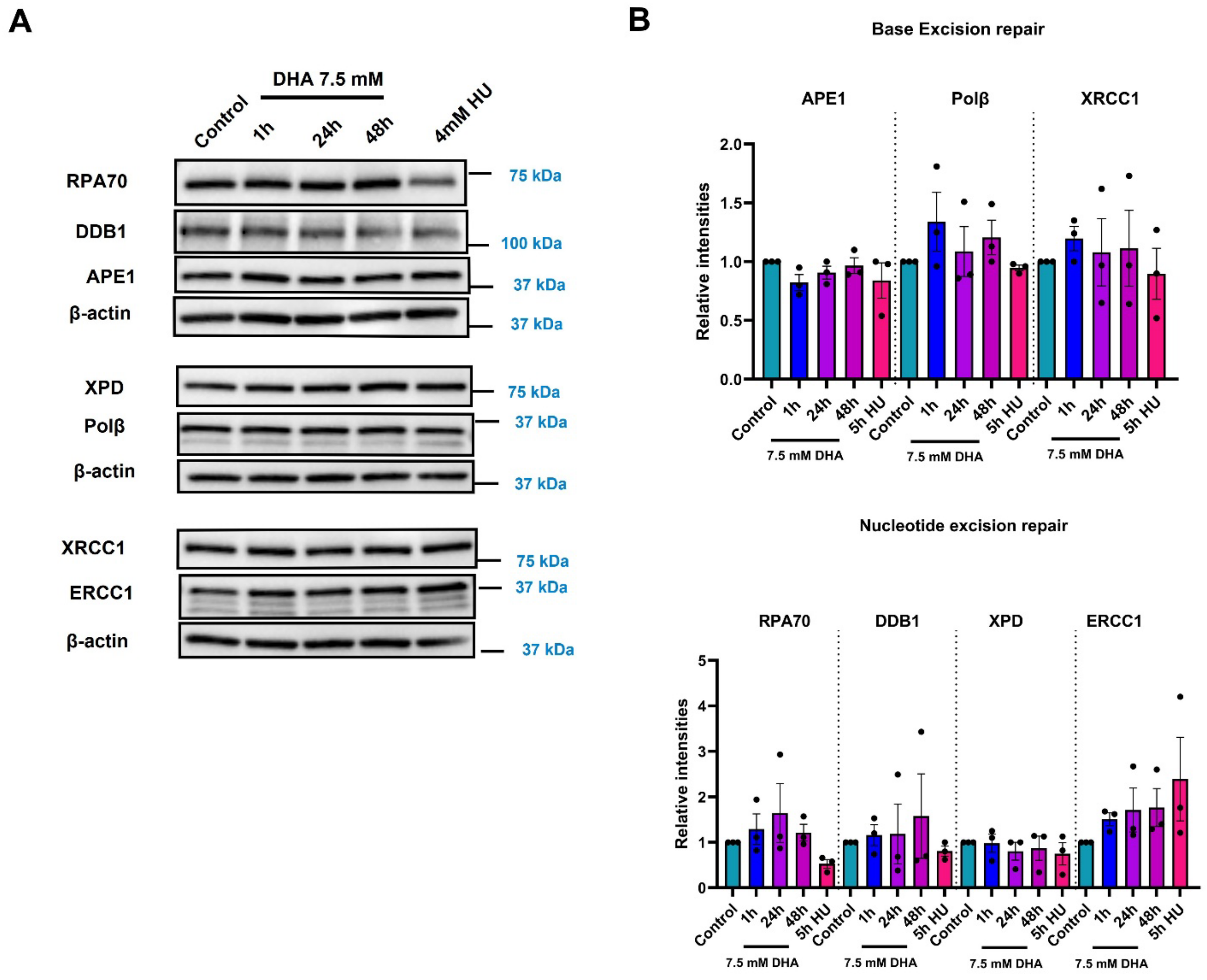


**Supporting Figure 2.** DNA repair pathways are intact in DHA exposed BEAS-2B cells. (A) Levels of base excision repair (BER), and (B) nucleotide excision repair (NER) pathway proteins probed in DHA treated cells at 1, 24 and 48 h after 7.5 mM DHA exposure. Hydroxyurea (HU) was used as a positive control. Cells were exposed to 4 mM HU for 5 h. Due to the overlapping size of some of the BER proteins, we used multiple membranes to probe the proteins presented. The membrane used to probe XRCC1 and ERCC1 is the same as the one used for p53 as shown in Figure 2C of the manuscript. These two gels have same β-actin loading control because they came from the same membrane. Graphs are displayed as the mean ± SEM over three biological replicates. The statistical difference was analyzed by one-way ANOVA coupled with Dunnett's post hoc test and displayed as follows: *$p<0.05$ and ****$p<0.0001$.

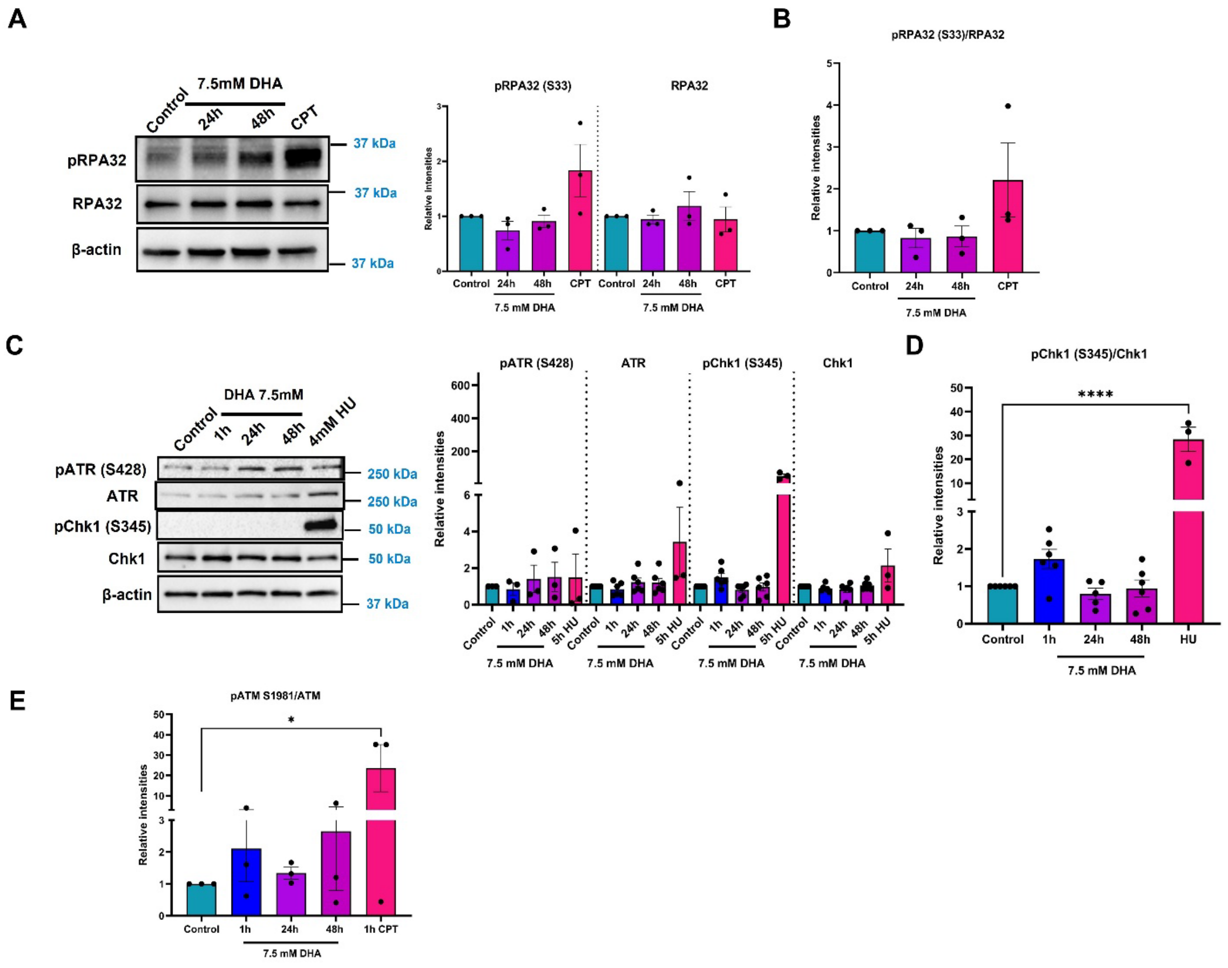


**Supporting Figure 3.** Examination of DNA damage response in BEAS-2B cells. Immunoblots showing (A) pRPA32 and total RPA32 protein levels and (B) their ratio in BEAS-2B cells exposed to 7.5 mM DHA for indicated times. CPT treatment at 100nM for 1 h was taken as positive control. (C) Protein levels of phosphorylated and total ATR and Chk1 protein and (D) ratio of pChk1/Chk1 in BEAS-2B cells exposed to 7.5mM DHA for indicated times. Exposure to 4mM HU for 5 h was taken as positive control. (E) Quantification of phosphorylated to total ATM ratio in DHA exposed BEAS-2B cells. Graphs are displayed as the mean ± SEM over three biological replicates. The statistical difference was analyzed by one-way ANOVA coupled with Dunnett's post hoc test and displayed as follows: *p<0.05 and ****p<0.0001.

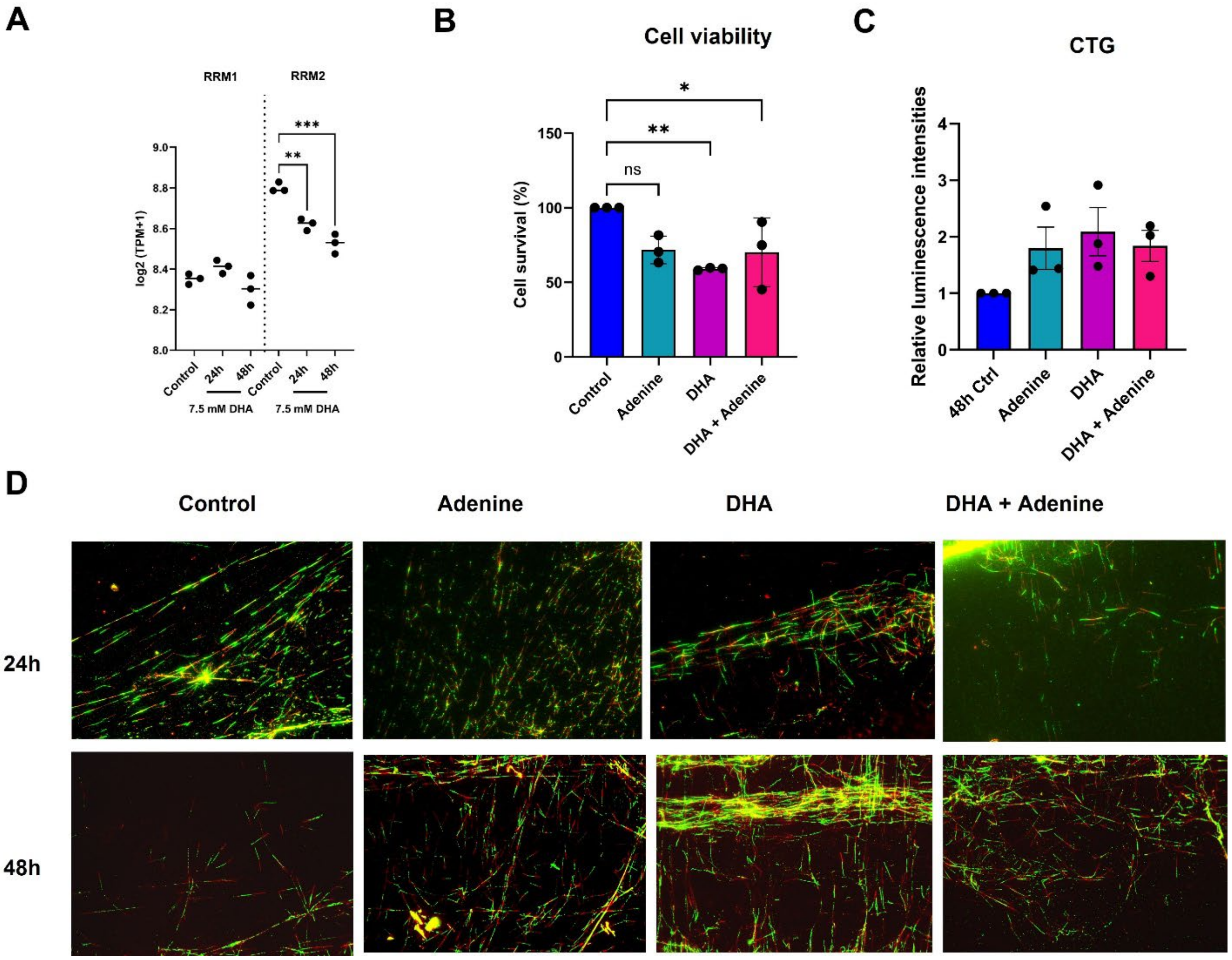


**Supporting Figure 4.** Transcript expression of RNR subunits, and impact of adenine supplementation on ATP levels and BEAS-2B cell survival (A) RRM1 and RRM2 transcript levels in control and DHA treated BEAS-2B cells. (B) Effects of 0.5 mM adenine supplementation in BEAS-2B cell viability after day 5 of exposure (C) Measurement of ATP contents using Cell titer glo (CTG) assay in adenine supplemented BEAS-2B cells. (D) Effect of adenine supplementation on replication fork as measured by DNA fiber assay at 24 and 48h. Graphs are displayed as the mean ± SEM over three biological replicates. The statistical difference was analyzed by one-way ANOVA coupled with Dunnett's post hoc test and displayed as follows: *p<0.05 and ****p<0.0001.

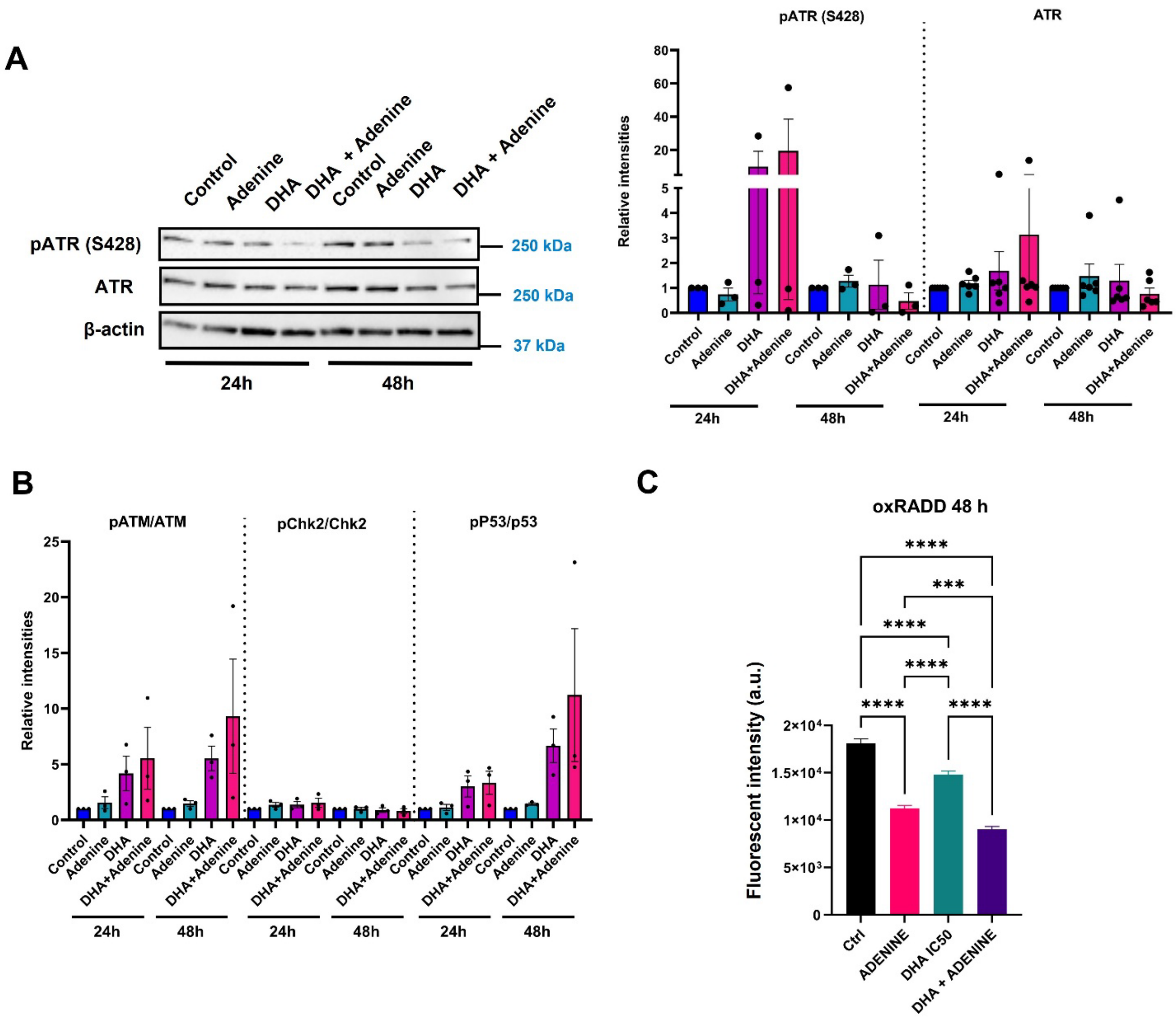


**Supporting Figure 5.** Impact of DHA treatment and adenine supplementation on DNA damage response and oxidative lesions in BEAS-2B cells. (A) Immunoblot analysis of ATR pathway protein after DHA and DHA plus adenine co-exposure. (B) Ratio of phosphorylated to total protein levels of ATM pathway protein in DHA and DHA plus adenine co-exposed BEAS-2B cells at indicated time-points. (C) Quantification of fluorescence intensities to measure oxidative lesions through RADD assay in DHA and DHA and adenine co-treated BEAS-2B cells at 48 h. The statistical difference was analyzed by one-way ANOVA coupled with Dunnett's post hoc test and displayed as follows: *$p<0.05$ and ****$p<0.0001$.
